# SRGN crosstalks with YAP to maintain chemoresistance and stemness in breast cancer cells by modulating HDAC2 expression

**DOI:** 10.7150/thno.41008

**Published:** 2020-03-04

**Authors:** Zhijie Zhang, Ni Qiu, Jiang Yin, Jianlei Zhang, Hao Liu, Wei Guo, Meijun Liu, Ting Liu, Danyang Chen, Kai Luo, Hongsheng Li, Zhimin He, Jinbao Liu, Guopei Zheng

**Affiliations:** 1 Affiliated Cancer Hospital & Institute of Guangzhou Medical University; Guangzhou Municipal and Guangdong Provincial Key Laboratory of Protein Modification and Degradation; The State Key Laboratory of Respiratory; Guangzhou Key Laboratory of "Translational Medicine on Malignant Tumor Treatment”, Hengzhigang Road 78#, Guangzhou 510095, Guangdong, China.; 2 Guangzhou Municipal and Guangdong Provincial Key Laboratory of Protein Modification and Degradation, School of Basic Medical Sciences, Guangzhou Medical University, Guangzhou, Guangdong, China.

**Keywords:** breast cancer, chemoresistance, SRGN, YAP, HDAC2

## Abstract

**Background**: Chemoresistance is a significant obstacle to the effective treatment of breast cancer (BC), resulting in more aggressive behavior and worse clinical outcome. The molecular mechanisms underlying breast cancer chemoresistance remain unclear. Our microarray analysis had identified the overexpression of a small molecular glycoprotein serglycin (SRGN) in multidrug-resistant BC cells. Here, we aimed to investigate the role of SRGN in chemoresistance of breast cancer and elucidate the underlying mechanisms.

**Methods**: SRNG overexpression was identified using microarray analysis and its clinical relevance was analyzed. To investigate the role of SRGN, we performed various *in vitro* and *in vivo* studies, as well as characterization of serum and tissue samples from BC patients. Chemosensitivity measurement, gene expression interference, immunofluorescence staining, mammosphere assay, flow cytometry analysis, luciferase reporter assay, ChIP-qPCR, coimmunoprecipitation, and immunohistochemistry were performed to explore the potential functions and mechanisms of SRGN.

**Results**: We confirmed overexpression of SRGN in chemoresistant BC cells and in serum and tissue samples from BC patients with poor response to chemotherapy. SRGN specifically predicted poor prognosis in BC patients receiving chemotherapy. Mechanistically, SRGN promoted chemoresistance both *in vitro* and *in vivo* by cross-talking with the transcriptional coactivator YES-associated protein (YAP) to maintain stemness in BC cells. Ectopic YAP expression restored the effects of *SRGN* knockdown. Inversely, YAP knockdown rescued the effects of *SRGN* overexpression. The secreted SRGN triggered ITGA5/FAK/CREB signaling to enhance *YAP* transcription. Reciprocally, YAP promoted *SRGN* transcription in a TEAD1-dependent manner to form a feed-forward circuit. Moreover, the YAP/RUNX1 complex promoted *HDAC2* transcription to induce chemoresistance and stemness in BC cells. Importantly, the SRGN levels were positively correlated with the YAP and HDAC2 levels in chemoresistant BC tissues. YAP and HDAC2 acted downstream of SRNG and correlated with poor outcomes of BC patients receiving chemotherapy.

**Conclusions**: Our findings clarify the roles and mechanisms of SRGN in mediating chemoresistance in breast cancer and suggest its use a potential biomarker for chemotherapeutic response. We believe that novel therapeutic strategies for breast cancer can be designed by targeting the signaling mediated by the crosstalk between SRGN and YAP.

## Introduction

Breast cancer (BC) is one of the most common malignancies in women. An estimated 2.1 million breast cancer cases were diagnosed worldwide in 2018, accounting for approximately 25% of cancer cases among women. Although the treatment of breast cancer has been greatly improved during the past decades, breast cancer deaths account for approximately 15% of total cancer deaths in women 1. Breast cancer presents as a heterogeneous cancer type with a variety of histopathological features and genetic markers and carries diverse prognostic outcomes. It can be divided into five major intrinsic subtypes based on gene expression profiling: luminal A, luminal B, ErbB2-enriched, triple-negative breast cancer (TN-BC), and a normal breast-like subtype 2-5. TN-BC is characterized by the lack of expression of the estrogen receptor (ER), progesterone receptor (PR) and epidermal growth factor receptor (HER2/ErbB2) and generally includes basal-like and claudin-low subtypes. According to the clinical subtype, therapeutic mainstays include endocrine therapy, anti-HER2 targeting, and chemotherapy. However, chemoresistance seems to be inevitable and subsequently leads to recurrence and metastasis.

Chemoresistance is a major obstacle to effective cancer treatment and almost always leads to metastatic progression and recurrence, resulting in a poor outcome 6 It includes intrinsic resistance to chemotherapy drugs prior to treatment and acquired resistance, especially de novo multidrug resistance, during treatment by cancers that were initially sensitive to chemotherapy 7, 8. Chemoresistance results from numerous biochemical changes mediated by genetics and epigenetics, including the increase in transporters that export anticancer drugs and the activation of anti-apoptotic and survival pathways, as well as the enrichment of cancer stem cells (CSCs) 9-11. Although the mechanisms of chemoresistance are being explored widely, they are still far from being fully understood. Therefore, identification of proteins and signaling pathways responsible for chemoresistance would contribute to the development of biomarkers for evaluating chemosensitivity and predicting patient prognosis, subsequently leading to the design of effective therapeutic strategies for breast cancer patients.

Serglycin (SRGN) is a low molecular weight glycoprotein that is distributed in cells as well as secreted and integrated into the extracellular matrix. SRGN plays an important role in the storage and secretion of many cytokines, chemokines, and proteases and is thus involved in many physiological and pathological processes 12, 13. Overexpression of SRGN has been reported in several types of cancers. In multiple myeloma, SRGN promotes tumor cell escape from immune surveillance by inhibiting complement activity 14, and its overexpression has been reported in the bone marrow aspirates of multiple myeloma patients 15. Increased levels of SRGN were found in the sera of hepatocellular carcinoma patients with bone metastasis 16. SRGN enhanced nasopharyngeal carcinoma (NPC) metastasis and was correlated with recurrence and poor prognosis in NPC patients 17. Overexpression of SRGN was detected in non-small lung cancers (NSCLCs), where it promoted aggressiveness by activating the CD44/NF-κB/CLDN1 axis and predicted poor outcomes in NSCLC patients 18.

SRGN was highly expressed in methotrexate- and vincristine-resistant hematopoietic tumor cells via unknown mechanisms, implying that SRGN might be involved in chemoresistance 19. Recently, we demonstrated that SRGN was overexpressed in TN-BC cells. SRGN induced TGFβ2 (transforming growth factor-β2) expression by activating CD44/ CREB1 signaling, and TGFβ2 induced SRGN expression by activating Smad3 to form a positive feedback loop, which in turn promoted epithelial-to- mesenchymal transition (EMT) to enhance TN-BC metastasis 20. These studies strongly suggested an important role of SRGN in human cancer progression; however, the precise role of SRGN and underlying molecular mechanisms in chemoresistance in breast cancer remained to be explored.

In our present study, we found that SRGN was overexpressed in chemoresistant BC cells, and its overexpression was involved in chemoresistance both *in vitro* and *in vivo*. Secreted SRGN mediated chemoresistance via the upregulation of the transcriptional coactivator YES-associated protein (YAP) expression by activating ITGA5/FAK/CREB signaling. Moreover, YAP positively regulated SRGN expression in a TEAD1-dependent manner to form a feed-forward circuit in chemoresistant BC cells. Additionally, YAP upregulated HDAC2 expression via the transcription factor RUNX1 to maintain stemness and chemoresistance in BC cells.

## Materials and Methods

### Cell culture and treatment

The BC cell lines MCF-7, T47D, and MDA-MB- 231 were obtained from ATCC. Cells were cultured in RPMI 1640 medium supplemented with 10% fetal bovine serum. The chemoresistant cell lines MCF-7/5- Fu and T47D/5-Fu were established from their isogenic cell lines MCF-7 and T47D, respectively, in our laboratory. Chemoresistant cells were established by intermittent stepwise selection *in vitro* with exposure to increased 5-Fu concentrations over a period of 12 months, starting at 1 mg/L and ending at 20 mg/L. The cell lines were cultured in the medium containing 2 μg/ml 5-Fu to maintain chemoresistance. To establish stable transfectants with knockdown or overexpression, cell lines were transfected with psi-LVRU6GP vectors containing shRNAs or with pEZ-SRGN lentiviral vectors overexpressing SRGN and were selected using puromycin.

### Patient samples

Sera and tumor tissue samples were collected from 25 BC patients each with good or poor response to chemotherapy at the Affiliated Cancer Hospital and Institute of Guangzhou Medical University. Serum samples were collected prior to any therapeutic procedures, such as chemotherapy and radiotherapy. This study was reviewed and approved by the Ethics Committees of Guangzhou Medical University and the Affiliated Cancer Hospital.

### Xenograft model in athymic mice

The animal studies were approved by the Institutional Animal Care and Use Committee (IACUC) of Guangzhou Medical University. Standard animal care and laboratory guidelines were followed according to the IACUC protocol. Cell lines were injected subcutaneously into the armpit of female BALB/c athymic nude mice to generate xenograft tumors (five mice per group). Ten days after cancer cell implantation, mice were injected intraperitoneally with 5-Fu or 5-Fu combined with VP. The treatment was administered every 3 days for 6 cycles. Tumor growth was measured every 2 days. The wet weight of the tumors was recorded after excision at the experimental endpoint.

The methods used in this study, including qRT- PCR, MTS assay, Western blotting, ELISA, immunofluorescence, mammosphere assay, flow cytometry analysis, luciferase reporter assay, chromatin immunoprecipitation (ChIP)-qPCR, coimmunoprecipitation, immunohistochemistry, and primers, are described in the Supplemental Experimental Procedures.

### Statistical Analysis

All data are presented as means ± s.d. Student's *t-*test was used to compare differences among different groups. Survival curves were plotted using the Kaplan-Meier method and compared using the log-rank test. Statistical analyses were performed using GraphPad Prism 6. *P* values of < 0.05 were considered statistically significant.

## Results

### Upregulation of SRGN is involved in chemoresistance in breast cancer cells

**Figure 1:**
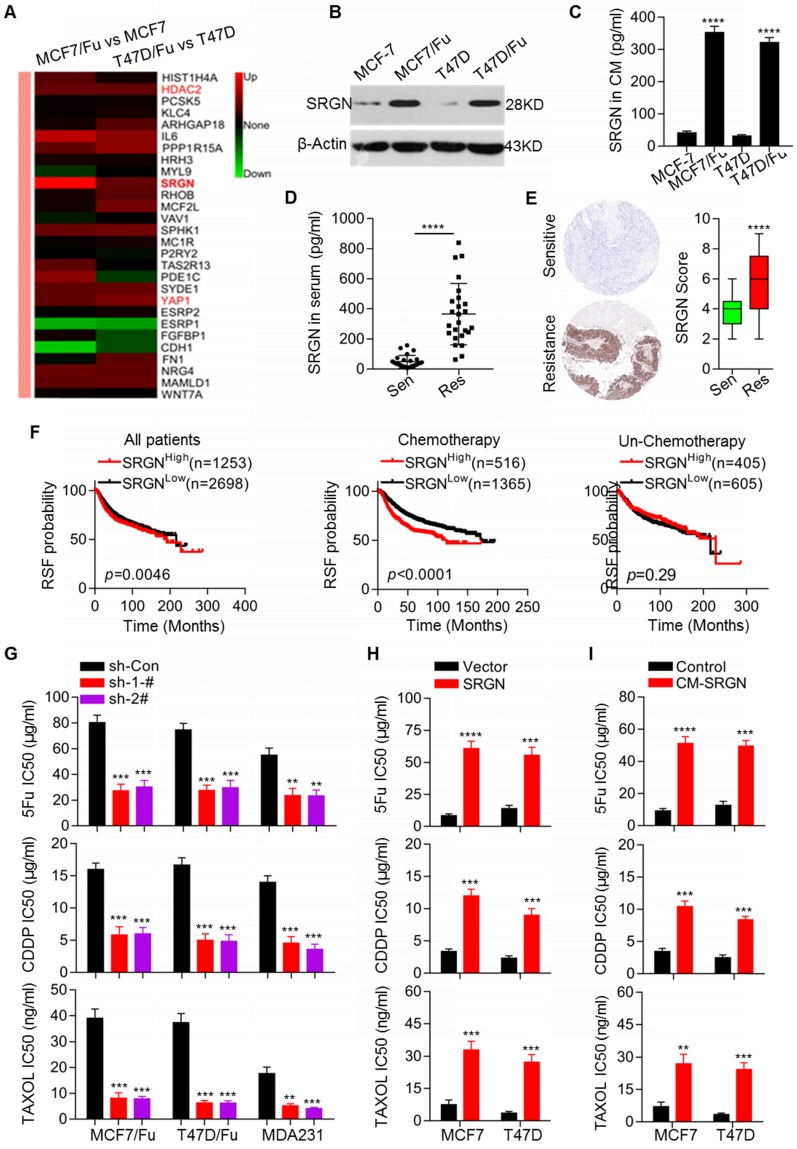
** Upregulation of SRGN is involved in mediating chemoresistance in breast cancer cells**. (A) Heatmap showing differentially expressed genes in chemoresistant BC cells compared to parental cells. (B) Western blot analysis of SRGN in the indicated cell lines. (C) SRGN protein levels in cell culture medium (CM) were detected by ELISA. Student's t-test; mean ± s.d. (n=3), _****_ *p* < 0.0001. (D) SRGN protein levels in sera from BC patients were measured by ELISA. Student's t-test, _****_ *p* < 0.0001. (E) SRGN protein levels in BC tissues were examined using immunohistochemistry, and expression scores were calculated. Student's t-test, _****_ *p* < 0.0001. (F) Kaplan-Meier plots by SRGN expression were generated for breast cancer patient cohorts in the TCGA database. Log-rank *p* values are shown. (G) IC50 values of drugs in the indicated cell lines with SRGN knockdown were calculated by the MTS assay. Student's t-test; mean ± s.d. (n=3), _**_ *p* < 0.01, _***_ *p* < 0.001. (H) IC50 values of drugs in the indicated cell lines with SRGN overexpression were calculated by the MTS assay. Student's t-test; mean ± s.d. (n=3), _***_ *p* < 0.001, _****_ *p* < 0.0001. (I) The IC50 values of drugs in the indicated cell lines treated with CM-SRGN were calculated the MTS assay. Student's t-test; mean ± s.d. (n=3), _**_ *p* < 0.01, _***_ *p* < 0.001, _****_ *p* < 0.0001.

To determine the molecular mechanisms underlying chemoresistance in BC, we established two chemoresistant BC cell lines, MCF-7/5-Fu and T47D/5-Fu derived from MCF-7 and T47D cell lines, respectively. The MCF-7/5-Fu and T47D/5-Fu cell lines showed significant resistance to 5-Fu, CDDP and Taxol (Figure S1A). We performed microarray analysis to screen differentially expressed transcripts of genes involved in chemoresistance between chemoresistant and parental cells. The heatmaps clearly showeddistinct expression patterns in parental and resistant cells (Figure S1B). A total of 822 differentially expressed genes were identified in both MCF-7/5-Fu and T47D/5-Fu cells (Figure S1C). Subsequently, a series of differentially expressed genes were selected for validation by qRT-PCR (Figure S1D). Among the annotated transcripts, the highly expressed SRGN transcript in both resistant cell lines attracted our attention (Figure 1A). The upregulation of SRGN mRNA and protein expression in resistant cell lines was validated (Figure 1B and Figure S1D). We also measured the absolute amounts of secreted SRGN in the culture medium (CM) of relevant cell lines by ELISA. The SRGN protein level in the CM of chemoresistant cells was much higher than that in the CM of parental cells (Figure 1C). Subsequently, we investigated the clinical relevance of SRGN in breast cancer. We measured SRGN protein levels in sera and tissue specimens collected from 25 BC patients each with good and poor response to chemotherapy. The protein levels of SRGN in serum from the group of patients with good response were much lower than those in serum from the group of patients with poor response (Figure 1D).

Consistent with this observation, the tissue specimens from the group of patients with good response had lower SRGN protein levels (Figure 1E). Next, we analyzed the association between the SRGN level and prognosis in BC patients using an online service (http://www.kmplot.com) and generated Kaplan- Meier plots. Our analysis demonstrated that in the total patient cohort, the SRGN level was negatively associated with RSF (Figure 1F). Importantly, the analysis was performed after patients were filtered by chemotherapy status to indicate the real prognostic value of SRGN expression. Notably, the SRGN level was not informative for the prognosis of BC patients without chemotherapy, but it predicted significantly unfavorable prognosis in BC patients receiving chemotherapy (Figure 1F).

To assess the involvement of SRGN upregulation in chemoresistance, its expression in chemoresistant cells was stably knocked down via transfection with SRGN-specific shRNAs resulting in reduced SRGN protein levels in the CM (Figure S1E). SRGN knockdown significantly enhanced the sensitivity of the chemoresistant cell lines MCF-7/5-Fu and T47D/5-Fu to chemotherapeutic agents, including 5-Fu, CDDP, and Taxol (Figure 1G). To confirm the effect of SRGN in BC cells, we also knocked down its expression in MDA-MB-231 cells with high endogenous expression levels of the protein 20 (Figure S1E). resulting in enhanced chemosensitivity of the cells (Figure 1G). Conversely, we overexpressed SRGN in MCF-7 and T47D cell lines (Figure S1F). As expected, after SRGN overexpression, its protein levels increased in the CM and the MCF-7 and T47D cell lines acquired resistance to chemotherapeutic drugs (Figure 1H) . To investigate whether SRGN exerts its effect on chemosensitivity in a secretion-dependent manner, the parental MCF-7 and T47D cells were incubated with CM from the corresponding SRGN-overexpressing cell lines (CM-SRGN). As shown in Figure 1I, incubation with CM-SRGN induced resistance to chemotherapeutic agents. Taken together, these data indicate that SRGN overexpression in chemoresistant breast cancer cells promotes chemoresistance and that SRGN level is negatively correlated with the prognosis of BC patients receiving chemotherapy.

### SRGN maintains breast cancer cell stemness by activating YAP signaling

**Figure 2:**
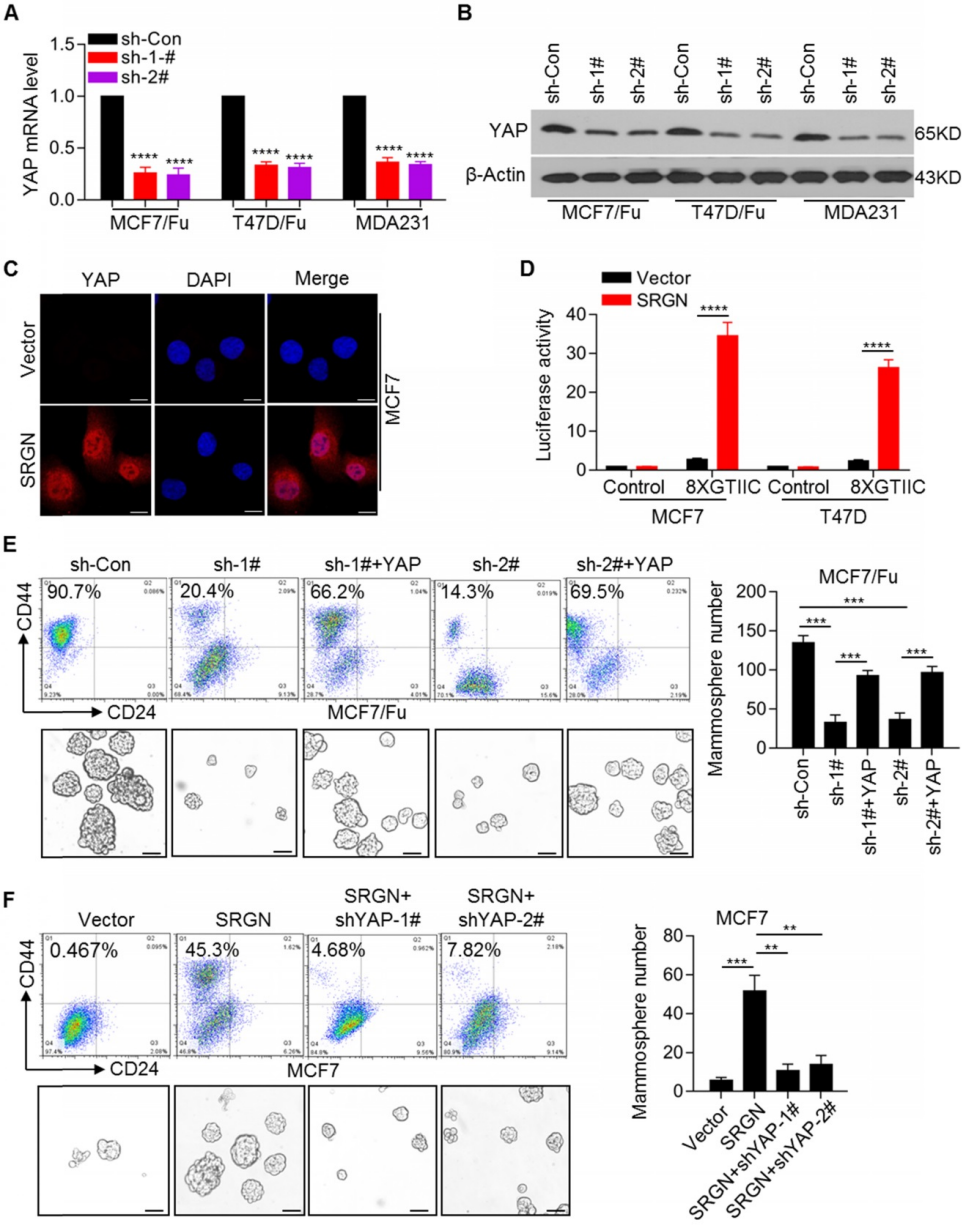
** SRGN maintains breast cancer stem cell traits by activating YAP signaling.** (A and B) YAP mRNA and protein levels in cell lines with SRGN knockdown were detected by qRT-PCR and Western blotting, respectively. Student's t-test; mean ± s.d. (n=3), _****_ *p* < 0.0001. (C) Immunofluorescence detection of YAP protein expression and nuclear translocation in MCF-7 cells with SRGN overexpression. Scale bar, 20μm. (D) The direct read-out of YAP transactivity was determined using a synthetic YAP/TAZ-responsive luciferase reporter (8XGTIIC-lux). Student's t-test; mean ± s.d. (n=3), _****_ *p* < 0.0001. (E) The percentage of CD44^high^/CD24^low^ cells was determined by flow cytometry and the mammosphere-forming ability was assessed in MCF-7/5-Fu cells with SRGN and YAP expression interference. Scale bar, 100 μm. Student's t-test; mean ± s.d. (n=3), _***_ *p* < 0.001. (F) The percentage of CD44^high^/CD24^low^ cells was determined by flow cytometry and the mammosphere-forming ability was assessed in MCF-7 cells with SRGN and YAP expression interference. Scale bar, 100 μm. Student's t-test; mean ± s.d. (n=3), _**_ *p* < 0.01, _***_ *p* < 0.001.

Given the upregulation of SRGN expression, which is involved in chemoresistance of BC cells, it was important to identify the downstream executors of SRGN-triggered signaling. Among the differentially expressed genes, YAP was highly expressed in chemoresistant cells (Figure 1A). We confirmed the upregulation of YAP mRNA and protein expression in chemoresistant BC cells (Figure S2A). The expression of the classical YAP downstream target genes, *CTGF* and *CYR61,* was also upregulated in chemoresistant BC cells (Figure S2B). As expected, SRGN knockdown using specific shRNAs robustly decreased the YAP mRNA and protein levels in MCF-7/5-Fu and T47D/5-Fu cell lines as well as in MDA-MB-231 cells with endogenous high SRGN expression (Figure 2A and 2B). SRGN knockdown also decreased the expression of the YAP target genes *CTGF* and *CYR61* (Figure S2C). In contrast, ectopic overexpression of SRGN significantly increased the YAP mRNA and protein levels and increased the expression of the YAP target genes, *CTGF* and *CYR61,* in the MCF-7 and T47D cell lines (Figure S2D). Subsequent immunofluorescence staining confirmed that SRGN positively regulated YAP protein expression and nuclear translocation (Figure 2C and Figure S2E). Moreover, we used a synthetic YAP/ TAZ-responsive luciferase reporter (8XGTIIC-lux) to determine the direct read-out of YAP transactivation and demonstrated that SRGN overexpression promoted the transactivation of YAP (Figure 2D). To investigate the role of YAP in BC cell lines, it was knocked down using shRNAs (Figure S3A), which reversed the chemoresistance of BC cells (Figure S3B). The acquisition of cancer stem cell (CSC) traits is usually correlated with therapeutic resistance and relapse 21. We observed an increase in the CD44^high^/CD24^low^ cell subpopulation and mammosphere-forming ability in chemoresistant BC cells (Figure S3C). To determine whether YAP affected SRGN in BC cells, we overexpressed YAP in chemoresistant BC cells in which SRGN was stably knocked down. Our results indicated that SRGN knockdown reduced the CD44^high^/CD24^low^ cell subpopulation and mammosphere-forming ability (Figure 2E), whereas the restoration of YAP expression abolished the effects of SRGN knockdown (Figure 2E). Consistent with these findings, the effects of SRGN on the YAP-mediated CD44^high^/CD24^low^ subpopulation enrichment and mammosphere- forming ability were confirmed in MDA-MB-231 cells with high endogenous expression of SRGN (Figure S3D). Also, ectopic SRGN expression enhanced the CD44^high^/CD24^low^ subpopulation and mammosphere- forming ability in MCF-7 cells (Figure 2F), but the YAP knockdown abolished the effects of SRGN overexpression (Figure 2F). These data indicated that SRGN maintained the status of breast cancer stem cell to mediate chemoresistance by enhancing YAP expression.

### SRGN enhances YAP expression by activating ITGα5/FAK/CREB signaling

Because SRGN is a secreted protein that positively regulates YAP expression at the transcriptional level, we next sought to identify the transcription factor that acts downstream of SRGN-triggered signaling to regulate YAP transcription directly. Previously, CREB was reported to transcriptionally regulate YAP expression 22.

**Figure 3:**
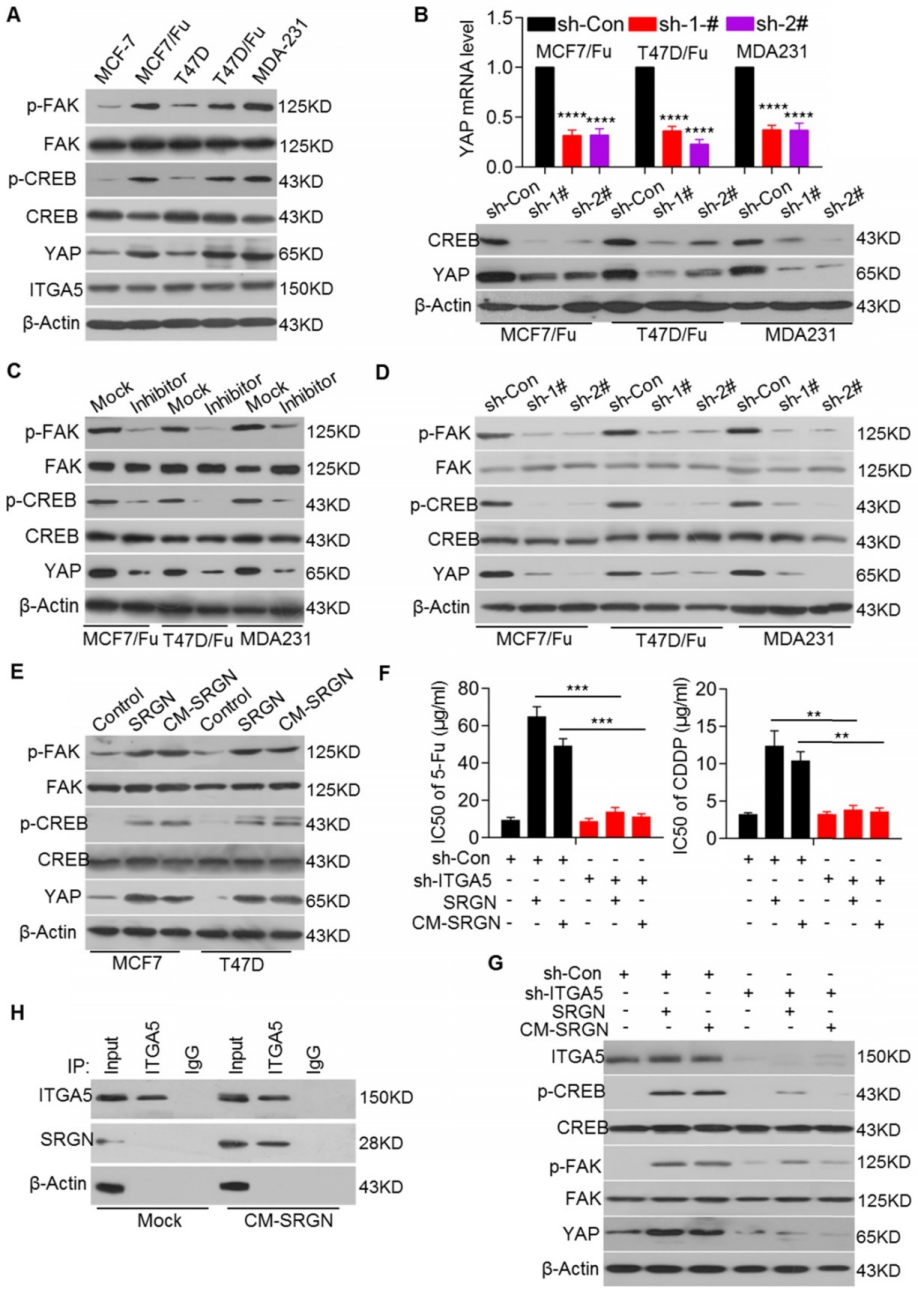
** SRGN enhances YAP expression by activating ITGα5/FAK/CREB signaling.** (A) Relevant protein levels in selected cell lines were assessed by Western blotting. (B) qRT-PCR and Western blotting were used to measure YAP mRNA (upper) and protein (lower) levels in cell lines with CREB knockdown. Student's t-test; mean ± s.d. (n=3), _****_ *p* < 0.0001. (C) Relevant protein levels in cell lines treated with a FAK inhibitor were examined by Western blotting. (D) Relevant protein levels in cell lines with SRGN knockdown were examined by Western blotting. (E) Relevant protein levels in cell lines overexpressing SRGN or incubated with CM-SRGN were examined by Western blotting. (F) IC50 values of drugs in MCF-7 cells with ITGA5 expression interference and either overexpressing SRGN or incubated with CM-SRGN were calculated by the MTS assay. Student's t-test; mean ± s.d. (n=3),_ **_ *p* < 0.01, _***_ *p* < 0.001. (G) Relevant protein levels in MCF-7 cells with ITGA5 expression interference and either overexpressing SRGN or incubated with CM-SRGN were examined by Western blotting. (H) Interaction between SRGN and ITGA5 was detected by a Co-IP assay.

Here, we investigated whether YAP was also regulated by CREB in BC cells. We demonstrated that chemoresistant BC and MDA-MB-231 cell lines with high endogenous expression of SRGN and YAP had higher p-CREB levels than the corresponding parental cell lines, although there was no difference in the total CREB protein levels (Figure 3A). As expected, CREB knockdown reduced YAP expression at both the mRNA and protein levels in chemoresistant BC cell lines (Figure 3B). We also found that FAK, upstream of CREB, was activated in chemoresistant BC and MDA-MB-231 cell lines, as evidenced by the increased p-FAK levels (Figure 3A). Treatment with the FAK inhibitor PF-573228 significantly decreased p-FAK and p-CREB levels and YAP mRNA and protein levels (Figure 3C and Figure S4A), suggesting that YAP expression is regulated by FAK/CREB signaling in chemoresistant BC cells.

Next, we investigated whether SRGN regulated the activation of FAK/CREB signaling. We found that SRGN knockdown in the MCF-7/5-Fu, T47D/5-Fu and MDA-MB-231 cell lines reduced the p-FAK and p-CREB levels, accompanied by a decrease in the YAP mRNA and protein levels but not by a change in the total FAK and CREB protein levels (Figure 3D and Figure 2A). Conversely, SRGN overexpression increased the p-FAK and p-CREB protein levels in the MCF-7 and T47D cell lines (Figure 3E). Since SRGN expression positively regulated FAK/CREB/YAP signaling, we sought to determine whether SRGN, as a secreted protein, modulated this signaling pathway in an autocrine or paracrine manner. We used CM from SRGN-overexpressing MCF-7 and T47D cells to culture MCF-7 and T47D cells, respectively. We found that CM-SRGN treatment induced an increase in the p-FAK and p-CREB protein levels, accompanied by an increase in the YAP mRNA and protein levels (Figure 3E and Figure S4B). These results implied that SRGN might activate FAK/CREB/YAP signaling via a cell membrane protein, which is expressed at a comparable level in both chemoresistant and the parental cell lines.

Previous studies have reported that integrins, as upstream regulators, could effectively activate FAK 23. Here, we found high endogenous expression of integrin alpha 5 (ITGA5) at comparable levels in selected BC cell lines (Figure 3A). To validate the role of ITGA5 in SRGN-mediated BC chemoresistance and FAK activation, ITGA5 was transiently knocked down in both chemoresistant and parental cell lines. ITGA5 knockdown enhanced the chemosensitivity of BC cells with high endogenous expression of SRGN (Figure S4C) but did not significantly affect BC cells with low endogenous expression of SRGN (data not shown). Additionally, ITGA5 knockdown abolished CM-SRGN-induced chemoresistance in MCF-7 cells (Figure 3F and Figure S4D), as well as SRGN overexpression- or CM-SRGN treatment-induced increases in the p-FAK and p-CREB protein levels and in the YAP mRNA and protein levels (Figure 3G and Figure S4E). Furthermore, the results of Co-IP assays demonstrated that extracellular SRGN could interact with ITGA5 after incubation of MCF-7 cells with SRGN-CM (Figure 3H). These data indicate that SRGN activates FAK/CREB/YAP signaling to mediate chemoresistance in an ITGA5-dependent manner in BC cells.

### A YAP-TEAD positive feedback loop regulates SRGN expression

**Figure 4:**
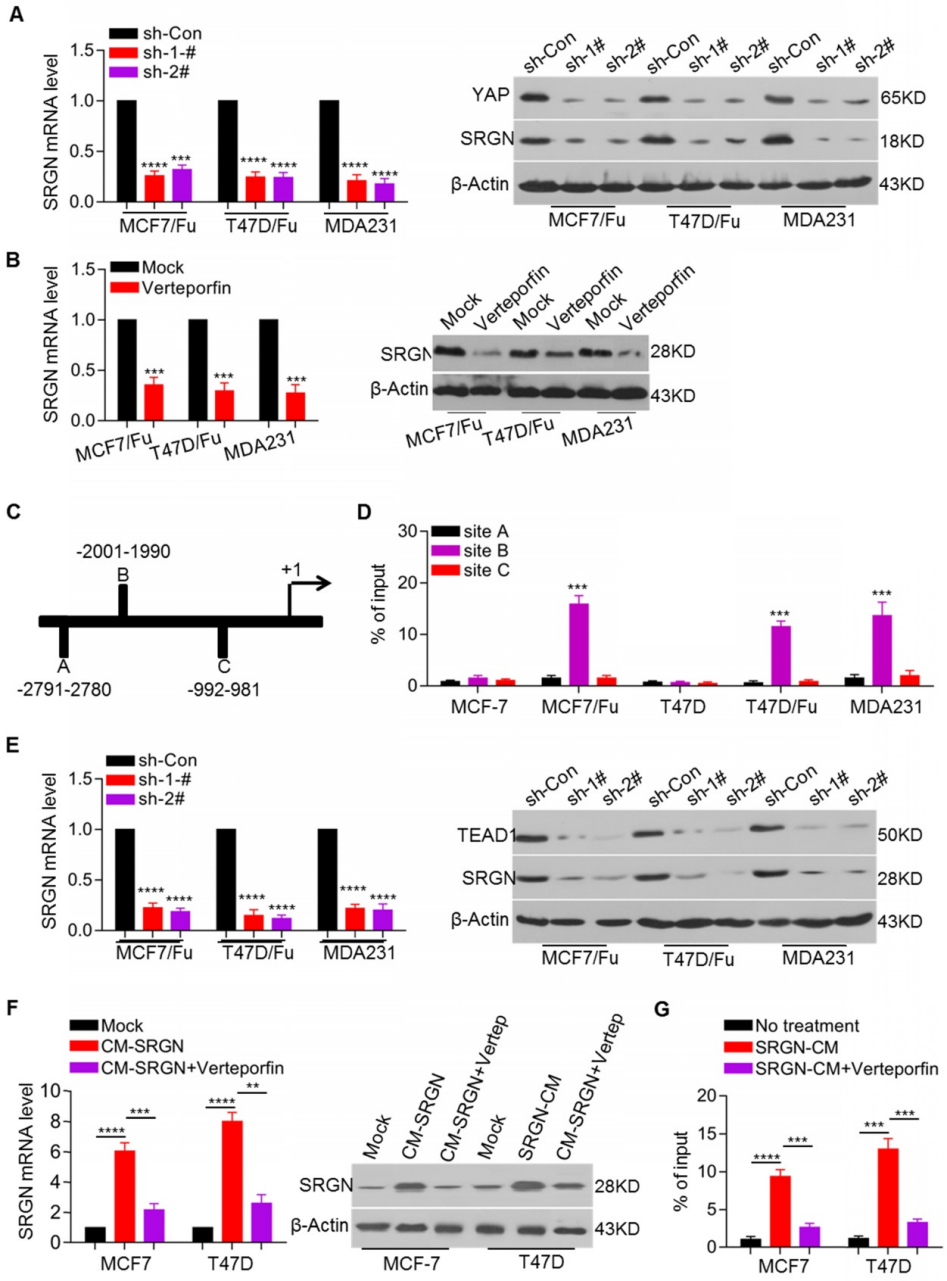
** YAP-TEAD positive feedback loop regulates SRGN expression.** (A) SRGN mRNA and protein levels in cell lines with YAP knockdown were examined by qRT-PCR and Western blotting, respectively. Student's t-test, mean ± s.d. (n=3), _***_ *p* < 0.001, _****_ *p* < 0.0001. (B) SRGN mRNA and protein levels in cells incubated with verteporfin (150 ng/ml) were examined by qRT-PCR and Western blotting, respectively. Student's t-test, mean ± s.d. (n=3), _***_ *p* < 0.001. (C) Putative TEAD1 binding site in the potential promoter region of *SRGN* was predicted by online analysis (http://jaspar.binf.ku.dk). (D) Enrichment of YAP in the *SRGN* promoter was determined by the ChIP assay. Student's t-test, mean ± s.d. (n=3), _***_ *p* < 0.001. (E) SRGN mRNA and protein levels in cell lines with TEAD1 knockdown were examined by qRT-PCR and Western blotting, respectively. Student's t-test, mean ± s.d. (n=3), _****_ *p* < 0.0001. (F) SRGN mRNA and protein levels in cells incubated with CM-SRGN and verteporfin were examined by qRT-PCR and Western blotting, respectively. Student's t-test, mean ± s.d. (n=3), _**_ *p* < 0.01, _***_ *p* < 0.001, _****_ *p* < 0.0001. (G) Enrichment of YAP in the *SRGN* promoter in cells incubated with CM-SRGN and verteporfin was determined by the ChIP assay. Student's t-test, mean ± s.d. (n=3), _***_ *p* < 0.001.

In view of the increased expression and important role of SRGN in chemoresistant BC cells and the involvement of regulatory feedback loops in cancer, we sought to determine whether SRGN- regulated YAP could regulate SRGN expression via a feedback mechanism. Here, we demonstrated that the SRGN mRNA and protein levels were decreased after YAP knockdown in the MCF-7/5-Fu, T47D/5-Fu and MDA-MB-231 cell lines (Figure 4A). YAP, as a transcriptional coactivator without DNA-binding domains, usually binds transcription factors such as TEAD1 to modulate target gene expression. To investigate whether YAP regulated SRGN by interacting with TEAD, the YAP-TEAD interaction inhibitor verteporfin was used to treat MCF-7/5-Fu, T47D/5-Fu and MDA-MB-231 cell lines. Verteporfin treatment significantly downregulated the SRGN mRNA and protein levels (Figure 4B).

We also performed an online analysis to screen potential transcription factors located within a three- kilobase region upstream of the SRGN transcription start site and predicted three TEAD1 binding sites in the potential promoter (Figure 4C). ChIP assays indicated that YAP was mainly enriched at one of the three predicted binding sites. YAP enrichment in the SRGN promoter region was higher in the MCF-7/5-Fu, T47D/5-Fu and MDA-MB-231 cell lines than in the MCF-7 and T47D cell lines (Figure 4D). To confirm its role in the regulation of SRGN expression, TEAD1 was transiently knocked down in the MCF-7/5-Fu, T47D/5-Fu and MDA-MB-231 cell lines resulting in a decrease in the SRGN mRNA and protein levels (Figure 4E). Also, endogenous SRGN expression was increased in the MCF-7 and T47D cell lines after incubation with CM-SRGN, which was abolished by treatment with the YAP-TEAD interaction inhibitor verteporfin (Figure 4F). Besides, CM-SRGN incubation significantly enhanced the enrichment of YAP in the SRGN promoter, which was prevented by TEAD1 knockdown (Figure 4G). These data indicate that YAP binds to the SRGN promoter via TEAD1 and promotes SRGN transcription, resulting in the formation of a positive feedback regulatory loop in BC cells.

### SRGN enhances HDAC2 expression to maintain CSC traits in BC cells

Since the ectopic overexpression of SRGN and incubation with CM-SRGN promoted chemoresistance in BC cells and the YAP-TEAD1 complex regulated SRGN expression via positive feedback, we sought to determine whether SRGN also regulated the YAP-mediated chemoresistance and cancer stem cell (CSC) status via YAP-TEAD1 interaction. When SRGN was ectopically overexpressed in MCF-7 and T47D cells with TEAD1 knockdown, and we found that YAP expression was upregulated and chemoresistance in MCF-7 and T47D cells was enhanced (Figure S5A). However, TEAD1 knockdown did not abolish the effect of SRGN overexpression on chemoresistance (Figure S5B). Similarly, the enhanced chemoresistance induced by CM-SRGN incubation was not weakened in MCF-7 and T47D cell lines with TEAD1 knockdown (Figure S5A and S5B). These results implied that SRGN/YAP axis-induced chemoresistance was not dependent on TEAD1.

**Figure 5:**
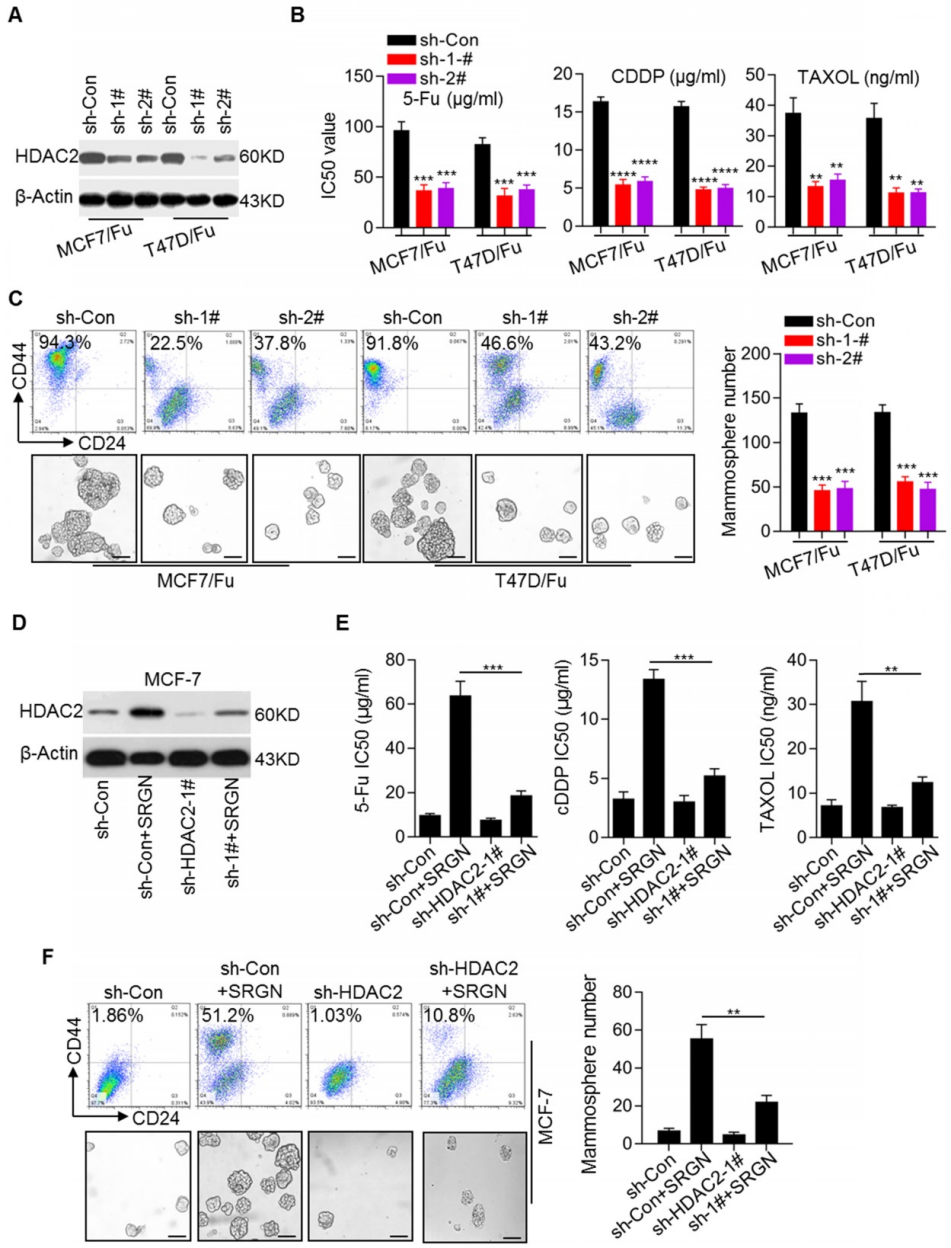
** SRGN enhances HDAC2 expression to maintain SC traits in BC cells.** (A) Efficiency of HDAC2 knockdown in chemoresistant BC cells was validated by Western blotting. (B) IC50 values of drugs in cell lines with HDAC2 knockdown were calculated by the MTS assays. Student's t-test, mean ± s.d. (n=3),_ **_ *p* < 0.01, _***_ *p* < 0.001, _****_ *p* < 0.0001. (C) The CD44^high^/CD24^low^ subpopulation was examined by flow cytometry and the mammosphere-forming ability was assessed in cell lines with HDAC2 knockdown. Scale bar, 100 μm. Student's t-test, mean ± s. d. (n=3), _***_ *p* < 0.001. (D) HDAC2 protein expression was detected by Western blotting. (E) IC50 values of drugs in MCF-7 cells with HDAC2 knockdown and SRGN overexpression were calculated by the MTS assay. Student's t-test, mean ± s.d. (n=3),_ **_ *p* < 0.01, _***_ *p* < 0.001. (F) CD44^high^/CD24^low^ cell population was examined by flow cytometry and the mammosphere-forming ability was assessed in MCF-7 cells with HDAC2 knockdown and SRGN overexpression. Scale bar, 100 μm. Student's t-test, mean ± s.d. (n=3), _**_ *p* < 0.001.

Recent reports have suggested the critical role of epigenetic modifications, including histone modifications, in tumorigenesis, cancer progression, and cancer stem cell status 24, 25. Here, we noted that HDAC2 expression was increased in chemoresistant BC cell lines (Figure 1A). Western blotting results confirmed the increase in the HDAC2 protein level in the MCF-7/5-Fu and T47D/5-Fu cell lines (Figure S5C). To investigate the role of HDAC2 in BC chemoresistance, the HDAC2 inhibitor CAY10683 was used. We found that treatment with CAY10683 significantly reversed chemoresistance in MCF-7/5- Fu and T47D/5-Fu cell lines (Figure S5D). To further confirm the role of HDAC2 in BC chemoresistance, HDAC2 expression was stably knocked down in the MCF-7/5-Fu and T47D/5-Fu cell lines (Figure 5A), which clearly reversed the chemoresistance (Figure 5B). Also, HDAC2 knockdown reduced the subpopulation of CD44^high^/CD24^low^ cells and the mammosphere-forming ability (Figure 5C). Moreover, to determine whether HDAC2 was involved in SRGN-induced chemoresistance and CSC traits in BC cells, HDAC2 expression was knocked down in MCF-7 cells prior to SRGN overexpression. Interestingly, SRGN overexpression promoted HDAC2 expression in MCF-7 cells (Figure 5D), and HDAC2 knockdown (Figure 5E) and HDAC2 inhibitor CAY10683 (Figure S5E) abolished the promotive effect of SRGN overexpression on chemoresistance. HDAC2 knockdown also inhibited the development of the CD44^high^/CD24^low^ cell subpopulation and the mammosphere-forming ability induced by SRGN overexpression (Figure 5F). These results suggest that SRGN-induced YAP promotes chemoresistance and CSC traits in a manner dependent on HDAC2 expression.

### YAP interacts with RUNX1 to transcriptionally regulate HDAC2 expression in BC cells

**Figure 6:**
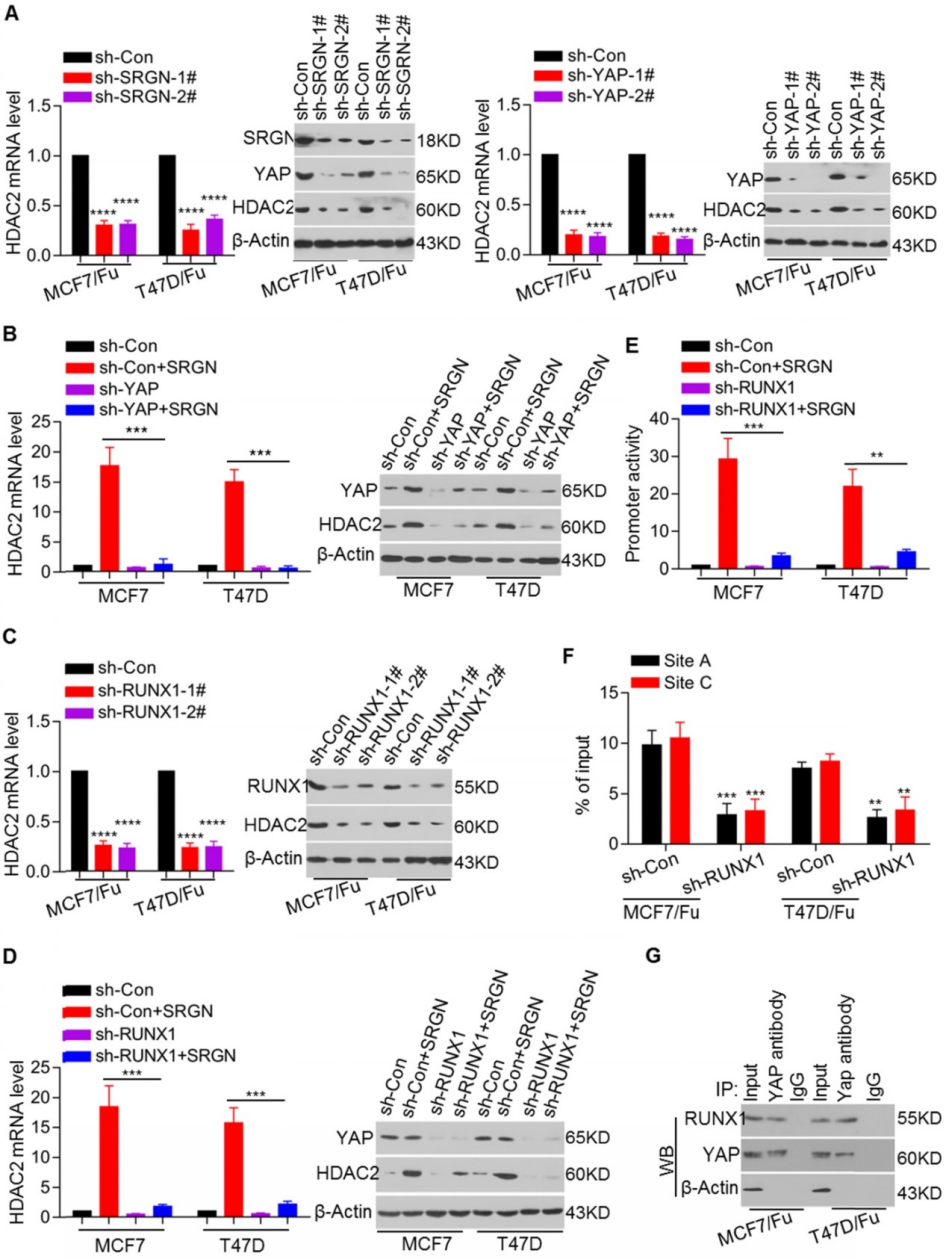
** YAP interacts with RUNX1 to transcriptionally regulate HDAC2 expression in BC cells.** (A) HDAC2 mRNA and protein levels in related cell lines with SRGN knockdown or YAP knockdown were examined by qRT-PCR and Western blotting, respectively. Student's t-test, mean ± s.d. (n=3), _****_ *p* < 0.0001. (B) HDAC2 mRNA and protein levels in cell lines with combined YAP knockdown and SRGN overexpression were examined by qRT-PCR and Western blotting, respectively. Student's t-test, mean ± s.d. (n=3), _***_ *p* < 0.001. (C) HDAC2 mRNA and protein levels in cell lines with TEAD1 knockdown were examined by qRT-PCR and Western blotting, respectively. Student's t-test, mean ± s.d. (n=3), _****_ *p* < 0.0001. (D) HDAC2 mRNA and protein levels in cell lines with combined TEAD1 knockdown and SRGN overexpression were examined by qRT-PCR and Western blotting, respectively. Student's t-test, mean ± s.d. (n=3), _***_ *p* < 0.001. (E) Luciferase activity driven by the HDAC2 promoter in cell lines with combined RUNX1 knockdown and SRGN overexpression was determined by a reporter assay. Student's t-test, mean ± s.d. (n=3),_ **_ *p* < 0.01, _***_ *p* < 0.001. (F) Enrichment of YAP at the HDAC2 promoter in cell lines with RUNX1 knockdown was detected by ChIP-qPCR. Student's t-test, mean ± s.d. (n=3),_ **_ *p* < 0.01, _***_ *p* < 0.001. (G) Interaction between YAP and RUNX1 was examined using a CoIP assay.

We showed that SRGN induced chemoresistance by regulating YAP expression and that SRGN maintained chemoresistance in a manner dependent on HDAC2 expression. Thus, it was important to explore the association between YAP and HDAC2. We demonstrated that knockdown of either SRGN or YAP decreased the HDAC2 mRNA and protein levels in the MCF-7/5-Fu and T47D/5-Fu cell lines (Figure 6A). To assess whether YAP directly mediated SRGN- induced HDAC2 expression, its expression was transiently knocked down in the MCF-7 and T47D cell lines with stable SRGN overexpression. The results indicated that SRGN induced HDAC2 expression in a YAP-dependent manner (Figure 6B). To confirm the role of TEAD1 in SRGN-induced HDAC2 expression, TEAD1 expression was transiently knocked down in the MCF-7 and T47D cell lines with stable SRGN overexpression. Interestingly, we found that SRGN overexpression-induced HDAC2 expression was independent of TEAD1 (Figure S6A). To identify transcription factors regulating HDAC2 expression, we used online software for the JASPAR database to analyze the response elements in a cohort of transcription factors located within the 3-kb region upstream of the HDAC2 transcription start site and found four putative RUNX1 binding sites (Figure S6B). Although there was no difference in RUNX1 expression between the chemoresistant cells and their corresponding parental cells (Figure S6C), RUNX1 knockdown led to a decrease in the HDAC2 mRNA and protein levels in MCF-7/5-Fu and T47D/5-Fu cells (Figure 6C). RUNX1 knockdown abolished the SRGN overexpression-induced HDAC2 upregulation (Figure 6D). When the 3-kb DNA sequence was cloned into the pGL4 reporter plasmid, the luciferase activity driven by the potential *HDAC2* promoter was much higher in MCF-7/5-Fu and T47D/5-Fu cells than in MCF-7 and T47D cells (Figure S6D). RUNX1, as well as YAP knockdowns, suppressed the HDAC2 promoter activity in MCF-7/5-Fu and T47D/5-Fu cells (Figure S6E). Additionally, RUNX1 knockdown inhibited the promoter activity induced by ectopic overexpression of SRGN in MCF-7 and T47D cells (Figure 6E). The ChIP-qPCR results revealed that YAP was mostly enriched at sites A and C within the *HDAC2* promoter and that the enrichment in MCF-7/5-Fu and T47D/5-Fu cells was much higher than that in MCF-7 and T47D cells lines (Figure S6F). Furthermore, RUNX1 knockdown resulted in a decrease in YAP enrichment at the HDAC2 promoter in MCF-7/5-Fu and T47D/5-Fu cells (Figure 6F), implying that RUNX1 was responsible for YAP enrichment at the HDAC2 promoter. The online server BioGRID was used to predict the interaction between YAP and RUNX1. Subsequently, we performed CoIP assays to confirm the interaction between YAP and RUNX1 in MCF-7/5-Fu and T47D/5-Fu cells (Figure 6G). Taken together, these results indicate that SRGN-induced YAP interacts with RUNX1 to transcriptionally regulate HDAC2 expression in BC cells.

### SRGN/YAP promotes chemoresistance *in vivo* and is correlated with poor outcomes in BC patients

**Figure 7:**
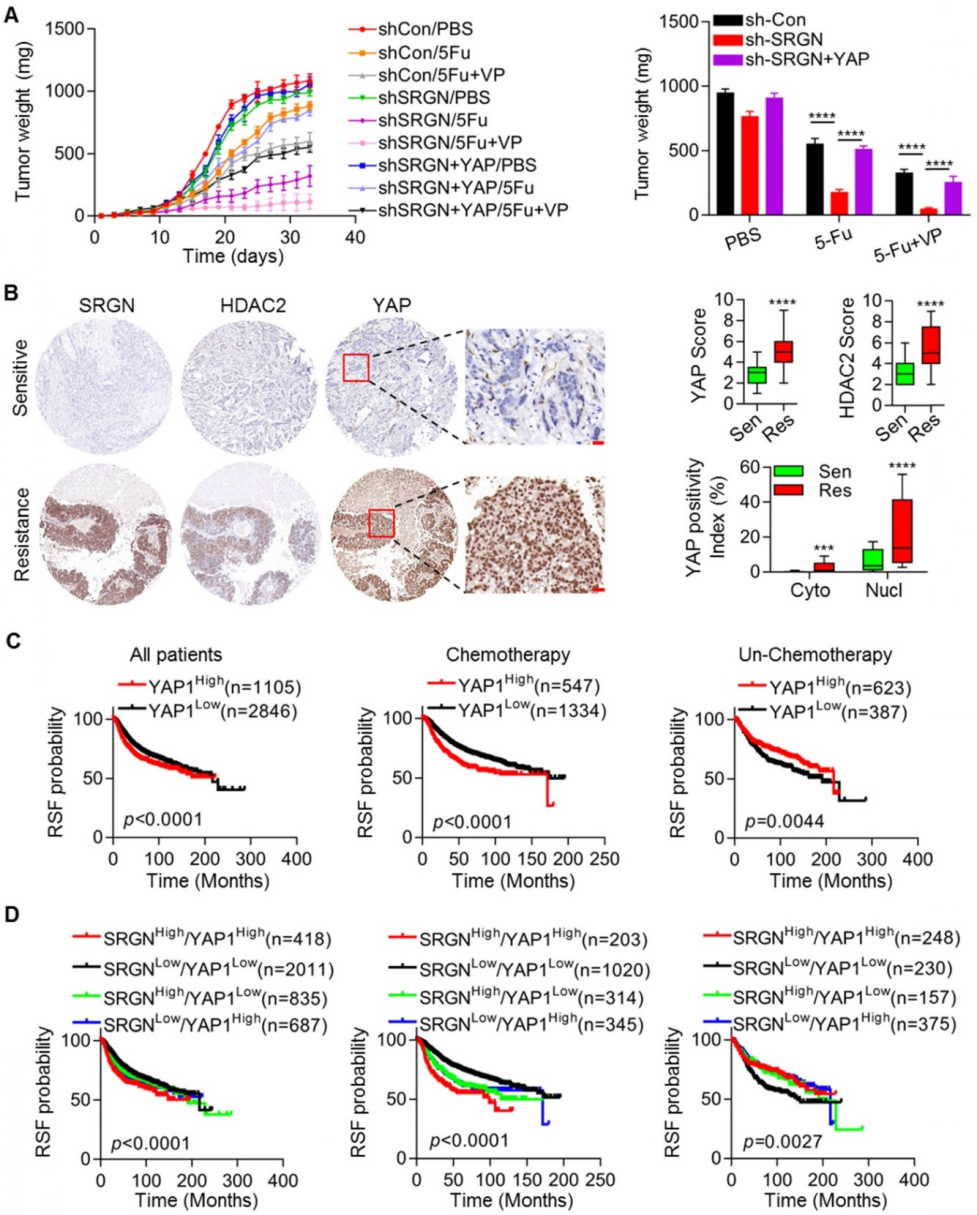
** SRGN/YAP promotes chemoresistance *in vivo* and is correlated with poor outcomes in BC patients.** (A) Growth and chemosensitivity were monitored in tumors derived from cells with combined SRGN knockdown and YAP overexpression. Tumor volumes were periodically measured for each mouse treated with 5-Fu alone or in combination with VP, tumor growth curves were plotted, and tumor wet weights were recorded. Student's t-test, mean ± s.d. (n=5/group), _****_ *p* < 0.0001. (B) Relevant protein levels in BC tissues were examined using immunohistochemistry, and expression scores were calculated. Student's t-test, _***_ *p* < 0.001, _****_ *p* < 0.0001. (C and D) Kaplan-Meier plots by SRGN and YAP expression were generated for BC patient cohorts in the TCGA database. Log-rank *p* values are shown.

Considering the important effects of SRGN/YAP signaling on BC chemoresistance *in vitro*, we further evaluated the biological role of the SRGN/YAP axis *in vivo*. We subcutaneously injected MDA-MB-231 cells with SRGN knockdown and control MDA-MB-231 cells into nude mice. SRGN knockdown significantly enhanced chemosensitivity. The tumors derived from cells with SRGN knockdown showed a longer initial response to chemotherapy than those derived from control cells. The volume and weight of the tumors derived from cells with SRGN knockdown were decreased to a significantly greater extent than those of control xenografts in response to 5-Fu alone and in combination with VP (Figure 7A and Figure S7A). However, restoration of YAP expression abolished the effect of SRGN knockdown on chemosensitivity (Figure 7A and Figure S7A). Additionally, the protein levels in the aforementioned tissues were assessed by immunohistochemical staining (Figure 1E). Tissue specimens with higher SRGN protein levels from patients with poor response also had higher YAP and HDAC2 protein levels than those from patients with good response (Figure 7B). The SRGN level was positively correlated with both the YAP and HDAC2 levels (Figure S7B) The YAP level was also positively correlated with the HDAC2 level (Figure S7B). To investigate whether treatment with chemotherapeutic drugs could induce SRGN secretion, the cellular localization of SRGN was investigated by flow cytometric analysis following drug treatment in MDA-MB-231 cells with high endogenous SRGN expression. As shown in Fig. S7C, the drugs induced the translocation of SRGN from the cytoplasm to the cell membrane and a significant increase in the SRGN protein level in the CM (Figure S7D).

We also analyzed the association between the SRGN/YAP/HDAC2 axis and prognosis in BC patients using the online resource and generated Kaplan-Meier plots. Our analysis demonstrated that in the total patient cohort, consistent with SRGN (Figure 1F), YAP and HDAC2 levels were negatively associated with RSF (Figure 7C and Figure S7E). Notably, when patients were filtered by chemotherapy status to indicate the prognostic value of SRGN signaling, the YAP level predicted a favorable prognosis in BC patients without chemotherapy (Figure 1F). However, the YAP and HDAC2 levels predicted unfavorable prognosis in BC patients receiving chemotherapy (Figure 7C and Figure S7E). Also, the BC patient group with high expression of both SRGN and YAP exhibited significantly worse prognosis than the group of patients with low expression of both SRGN and YAP, especially among BC patients receiving chemotherapy (Figure 7D). These data demonstrate a positive correlation between SRGN, YAP and HDAC2 expression in BC tissues. Our *in vivo* and clinical analysis supports the hypothesis that the SRGN/YAP/HDAC2 axis might play an important role in the chemotherapy response in BC patients.

## Discussion

**Figure 8:**
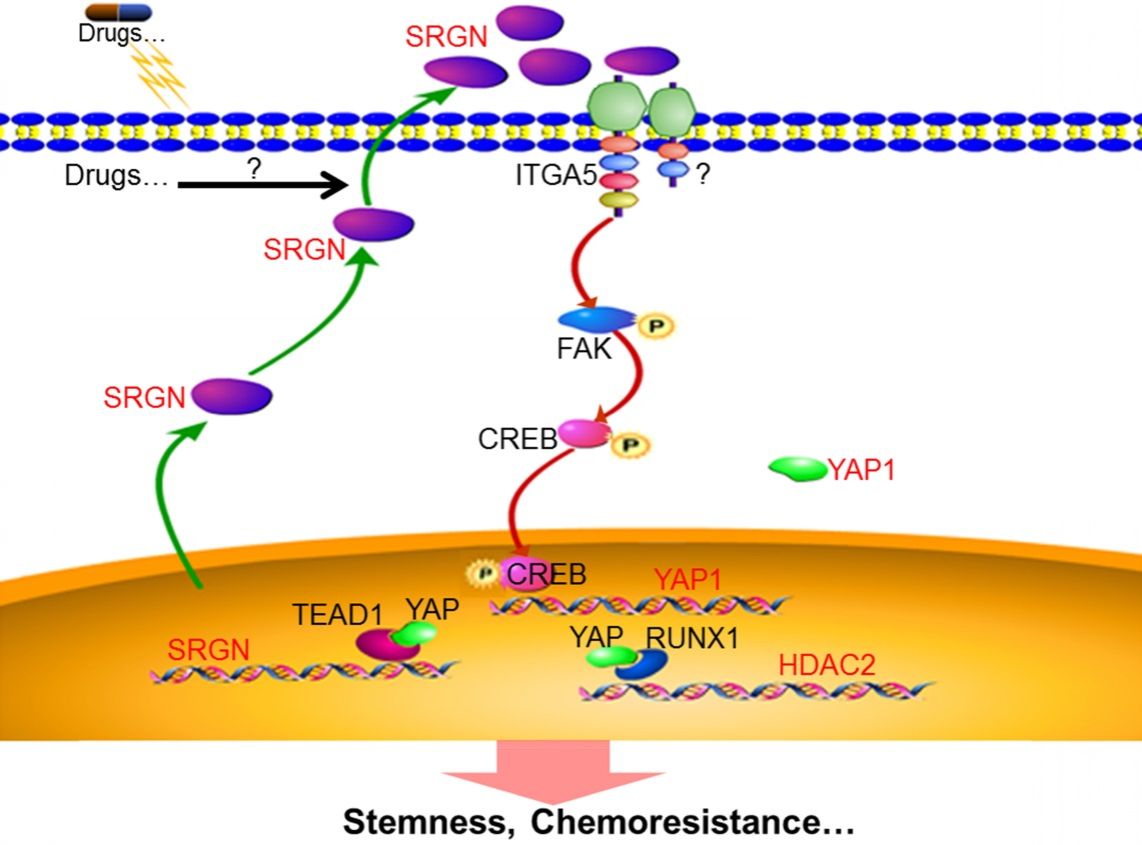
Schematic illustration of SRGN crosstalk with YAP to maintain chemoresistance and stemness in BC cells by modulating HDAC2.

In this study, we investigated the role of SRGN in BC cells using *in vitro* and *in vivo* models. Our findings provide new insights into the molecular mechanisms underlying chemoresistance in BC: (1) SRGN is overexpressed in chemoresistant cells, culture medium from chemoresistant cells and serum from BC patients with poor response to chemotherapy; (2) extracellular SRGN protein interacts with ITGA5 to activate FAK/CREB/YAP signaling; (3) YAP enhances SRGN expression dependent on TEAD1 to form a feed-forward circuit; and (4) YAP interacts with RUNX1 to upregulate HDAC2 expression to mediate chemoresistance (Fig. 8).

SRGN is a low molecular weight proteoglycan comprised of a core protein attached to negatively charged glycosaminoglycan (GAG) chains of either chondroitin sulfate or heparin 26. The core protein of SRGN includes 158 amino acid residues and contains three domains: a signal peptide and N-terminal and C-terminal domains. The detailed functions of these domains remain unclear 27. SRGN is widely distributed in cells as well as secreted and integrated into the extracellular matrix and is involved in many physiological and pathological processes 12, 13. SRGN expression has been examined in endothelial 28, hematopoietic 29 and embryonic stem cells 30. SRGN overexpression was found in a variety of cancers and is correlated with the development, progression, and aggressive biological behavior of tumors 14-18. We previously demonstrated that SRGN was highly expressed in TN-BC cells and was involved in metastasis 20. Since TN-BC cells show intrinsic therapeutic resistance, we aimed to investigate its possible correlation with SRGN. In this study, we showed high expression of SRGN in chemoresistant BC cells and the CM as well as in TN-BC cells. SRGN knockdown reversed chemoresistance, whereas SRGN overexpression enhanced chemoresistance. Moreover, incubation of BC cells with the CM from SRGN-overexpressing cells induced chemoresistance. Our results demonstrated that chemotherapy induced SRGN secretion by BC cells indicating that SRGN-mediated chemoresistance may occur via a secretion-dependent mechanism.

Although the involvement of SRGN in tumorigenesis has been investigated in many human cancers, the detailed mechanisms are still far from being fully understood. Here, we used reporter assays to identify the signaling pathway responsible for mediating the effect of SRGN-induced chemoresistance. We found that SRGN knockdown resulted in the downregulation of YAP mRNA and protein leading to decreased YAP signaling activity. Our data demonstrated that SRGN maintains the stemness and drug resistance of chemoresistant cells by enhancing YAP expression. YAP is the main downstream effector of the mammalian Hippo pathway and regulates tissue homeostasis and regeneration, organ development, and stem cell activation. Studies in transgenic mice, Hippo pathway knockout mice, and YAP knockout mice showed that YAP is required for adult stem cell activation during tissue damage, and that aberrant YAP activation expands epithelial stem/progenitor cells *in vivo* 31-34.

YAP is a potential oncogene, located in the 11q22 amplicon often amplified in various human cancers 35, and is tightly regulated post-transcriptionally by upstream kinase-mediated degradation or cytoplasmic sequestration, resulting in its shuttling between the nucleus and cytoplasm. In response to unfavorable extracellular or intracellular signals, MST1/2 phosphorylates and activates LATS1/2. Activated LATS1/2, in turn, phosphorylates YAP at serine 127, which is one of the most common posttranslational modifications resulting in the cytoplasmic localization of YAP1 to repress its activity 36. In the nucleus, YAP triggers downstream biological effects by inducing a transcription program through interacting with related transcription factors, especially TEADs, leading to an increase in cancer cell proliferation, cancer stemness, metastasis, and therapeutic resistance 37, 38. YAP has also been reported to be an oncogene in breast cancer and was involved in the induction of stemness in mammary epithelial cells and breast cancer 39. YAP activation was correlated with bone metastasis and unfavorable outcomes in breast cancer 40. Interestingly, we found here that, beyond posttranscriptional modification, SRGN regulated YAP expression at the transcriptional level.

Previous studies showed that CREB transcriptionally induced YAP expression and subsequently promoted growth of hepatoma cells *in vivo* and *in vitro* 22. Recently, we also showed that SRGN could activate CREB via CD44 and induce TGFβ2 expression in TN-BC cells 20. Here, we demonstrated that CREB was activated in chemoresistant cells compared to parental cells and that its knockdown reduced YAP expression. We also demonstrated that FAK, as an upstream regulator of CREB 41, was activated in chemoresistant cells and that inhibition of FAK activity reversed chemoresistance. Consistently, SRGN knockdown led to inhibition of FAK/CREB activity. SRGN overexpression or CM-SRGN treatment induced chemoresistance and FAK/CREB/YAP signaling activation in parental cells with low endogenous CD44 expression. Thus, we hypothesized that SRGN triggered FAK/CREB/YAP signaling via proteins expressed in both the chemoresistant and parental cells. Next, we focused on ITGA5 because it acts upstream of FAK 23. ITGA5 expression was comparable between chemoresistant cells and parental cells. As expected, ITGA5 knockdown abolished the SRGN overexpression- or CM-SRGN treatment-induced activation of FAK/CREB/YAP signaling and chemoresistance in parental cells. Incubation with CM-SRGN resulted in interaction between SRGN and ITGA5. Our results strongly supported the hypothesis that the SRGN-induced activation of FAK/CREB/YAP signaling to mediate chemoresistance is dependent on ITGA5.

To maintain signaling pathway activation, feed-forward regulatory loops operate in cancer cells. Here, we sought to determine whether YAP, as a transcriptional coactivator, could regulate SRGN expression to form a positive regulatory loop. We found that YAP knockdown reduced endogenous SRGN expression in chemoresistant cells. The bioinformatics analysis and experimental validation data confirmed that YAP transcriptionally regulated SRGN expression dependent on TEAD1. However, TEAD1 knockdown did not impair the effect of SRGN overexpression or CM-SRGN treatment on chemoresistance. Hence, we hypothesized that SRGN/YAP signaling induces chemoresistance via other mechanisms, and focused on epigenetic-related molecules such as HDACs (histone deacetylases) because of their critical roles in tumorigenesis, tumor progression, and therapeutic response 24, 42. Among the differentially expressed genes, HDAC2 was significantly upregulated in chemoresistant cells. SRGN induced or maintained breast cancer stemness and chemoresistance depending on the level of HDAC2. Thus, the bioinformatics analysis and experimental data supported that SRGN/YAP signaling transcriptionally regulated HDAC2 expression dependent on RUNX1 but not TEAD1. HDACs, in conjunction with histone acetyl transferases (HATs), determine the acetylation status of lysine residues on histones as an important chromatin modification to regulate gene expression. In this manner, HDACs play important roles in a variety of molecular events, such as DNA replication, gene transcription, DNA damage, DNA repair, protein stability, and in signaling pathways involved in biological processes such as proliferation, mitosis, differentiation, immune function, and circadian rhythms 25.

HDAC overexpression has been found in many human cancers and is correlated with cancer growth and poor prognosis 25. It was overexpressed in pancreatic ductal adenocarcinoma (PDAC) and conferred resistance to etoposide (a topoisomerase II inhibitor) in PDAC cells via the downregulation of the BH3-only protein NOXA 43. HDAC2 induced by beta-adrenergic signaling promoted tumor angiogenesis and prostate cancer progression by suppressing thrombospondin-1 expression 44. HDAC2 was found to be highly expressed in anaplastic thyroid cancer (ATC) and its functional inhibition repressed ATC growth and metastases 45. Recent studies showed that HDAC2 is involved in the maintenance of stem cell pluripotency. HDAC2 is essential for the self-renewal of embryonic stem cells (ESCs) through maintaining the expression of the key pluripotency transcription factors Oct4, Nanog, Rex1, and Esrrb 46. The SIN3A/HDAC corepressor complex maintained ESC pluripotency and promoted the generation of induced pluripotent stem cells (iPSCs) by SIN3A- and HDAC2-induced efficient reprogramming 47. HDAC activity has also been shown to be involved in the enrichment of cancer stem cells by chemotherapy and conferred chemoresistance 48. Consistent with these findings, we demonstrated that SRGN/YAP maintained the stemness of chemoresistant BC cells via HDAC2.

## Conclusion

Our work presented here delineates the role and mechanism of SRGN in BC chemoresistance. SRGN was upregulated in chemoresistant BC cells and was associated with poor clinical outcomes in BC patients receiving chemotherapy. Extracellular SRGN activated ITGA5/FAK/CREB/YAP signaling to form a positive feedback loop dependent on TEAD1 and to induce HDAC2 expression to maintain stemness and chemoresistance in BC cells. Our findings provide insights into SRGN-triggered signaling as a promising therapeutic target to reverse chemoresistance. Although our study has significant translational implications, further investigations are needed to precisely measure the SRGN protein level in serum to monitor the chemotherapeutic response and for targeting SRGN-triggered signaling to improve chemotherapeutic efficacy in BC patients.

## Supplementary Material

Supplementary figures and experimental procedures.
