# Supplementary material for: SRGN crosstalks with YAP to maintain chemoresistance and stemness in breast cancer cells by modulating HDAC2 expression
Source: Theranostics. 2020 Mar 4;10(10):4290–307. doi: 10.7150/thno.41008 (PMC7150493; doi:10.7150/thno.41008)
Supplement: Supplementary file 1 — Supplementary figures and experimental procedures. [file thnov10p4290s1.pdf]

Supplemental Information

SRGN crosstalks with YAP to maintain chemo-resistance and stemness of breast cancer cells via modulating HDAC2 expression

Supplementary Figure Legends

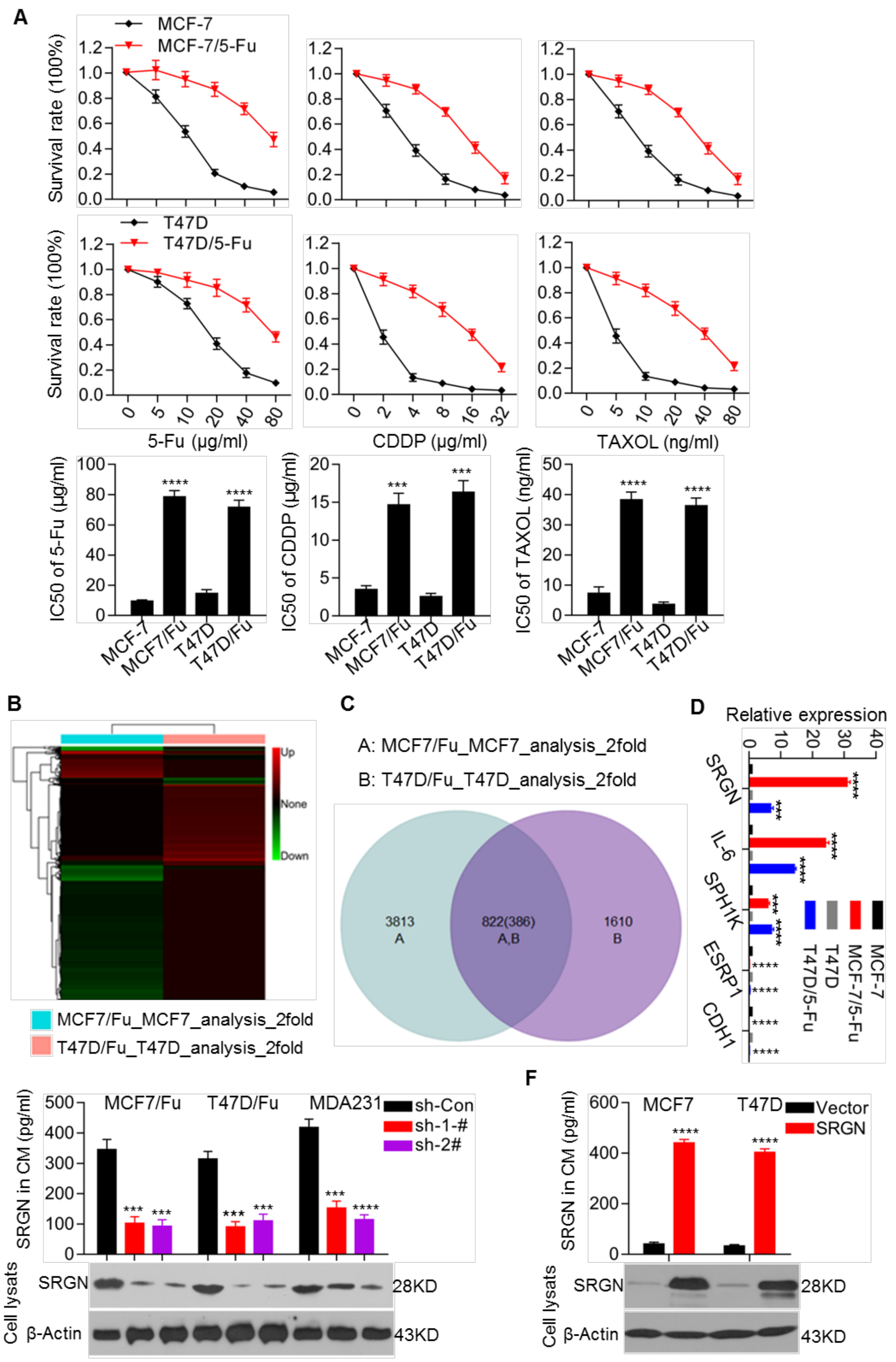

Figure S1, related to Figure 1. Up-regulation of SRGN is involved in chemo-resistance in breast cancer cells.

(A) The chemo-resistant characteristics of related cell lines was determined using

MTS assay. Dose-response curves of related cells to 5-Fu, cDDP or TAXOL were plotted and the IC50 values were calculated. Each point represents the mean of three independent experiments. (B and C) Differential genes expressed in chemo-resistant cells were analyzed using microarray analysis. (D) Some differential genes were selected for validation by qRT-PCR. (E) The effect of SRGN-specific shRNA transfection on SRGN protein levels in cells or in CM was detected by western blotting (lower) or ELISA (upper). Student's t-test, mean±s.d. (n=3), *** $p < 0.001$, **** $p < 0.0001$. (F) The SRGN protein levels in CM after SRGN overexpression were detected by ELISA (upper). The SRGN protein levels in cells after SRGN overexpression were detected by western blot (lower). Student's t-test, Mean±s.d. (n=3), *** $p < 0.001$, **** $p < 0.0001$.

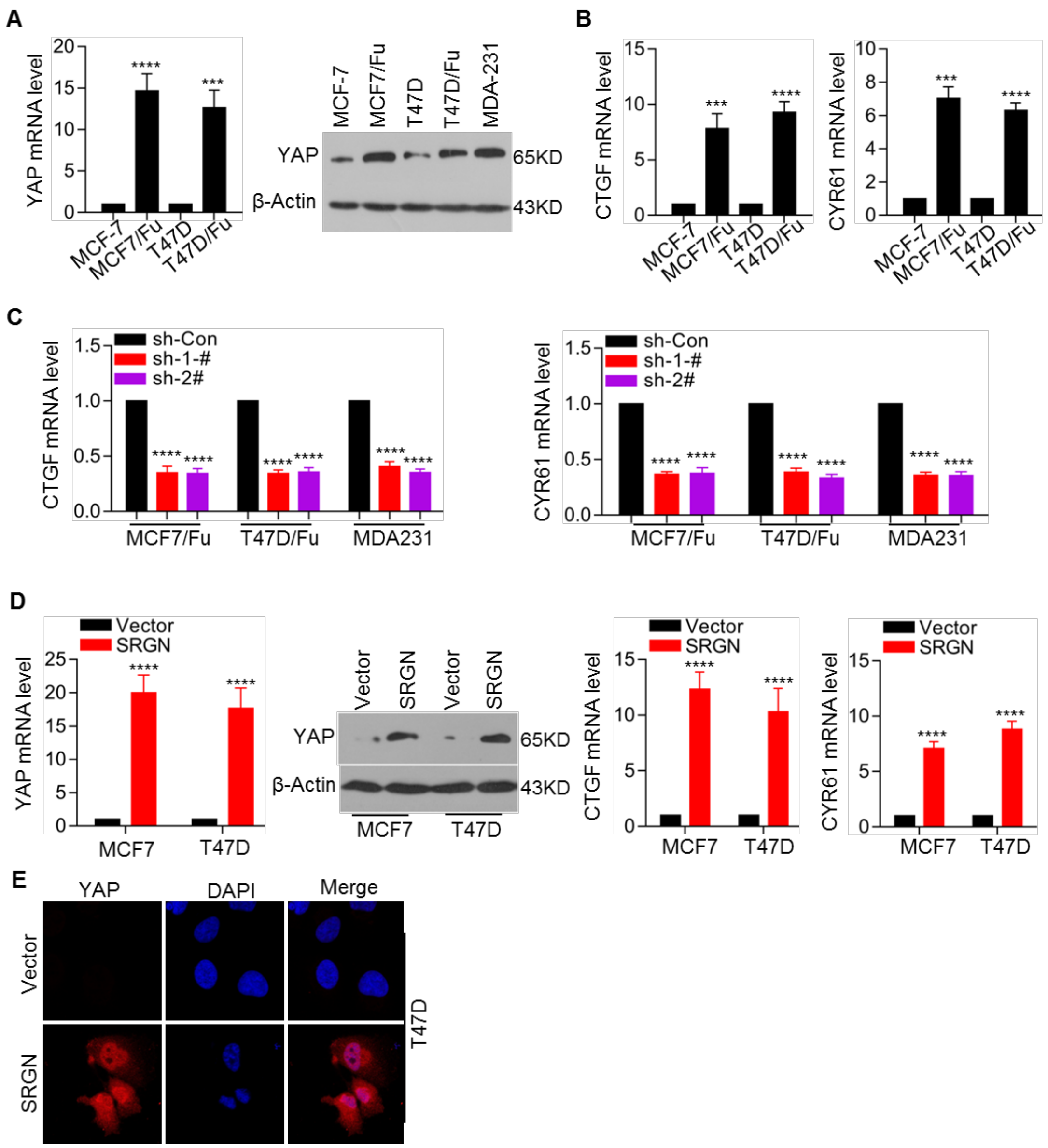

Figure S2, related to Figure 2. SRGN maintains breast cancer stem cell traits via activating YAP signaling.

(A) YAP mRNA and protein levels in cells were detected by qRT-PCR and western blot respectively. Student's t-test, Mean±s.d. (n=3), *** $p < 0.001$, **** $p < 0.0001$. (B) *CTGF* and *CYR61* mRNA levels in BC cells were determined by qRT-PCR. Student's t-test, Mean±s.d. (n=3), *** $p < 0.001$, **** $p < 0.0001$. (C) *CTGF* and *CYR61* mRNA levels in cell lines with SRGN knockdown were detected by qRT-PCR. Student's t-test, Mean±s.d. (n=3), **** $p < 0.0001$. (D) The YAP mRNA and protein levels in cell lines with SRGN overexpression were determined by qRT-PCR and western blotting,

respectively. *CTGF* and *CYR61* mRNA levels in cell lines with SRGN overexpression were detected by qRT-PCR. Student's t-test, Mean±s.d. (n=3), **** p < 0.0001. (E) Immunofluorescence detection of YAP protein level and nuclear translocation in T47D cells with SRGN overexpression. Scale bar, 20μm.

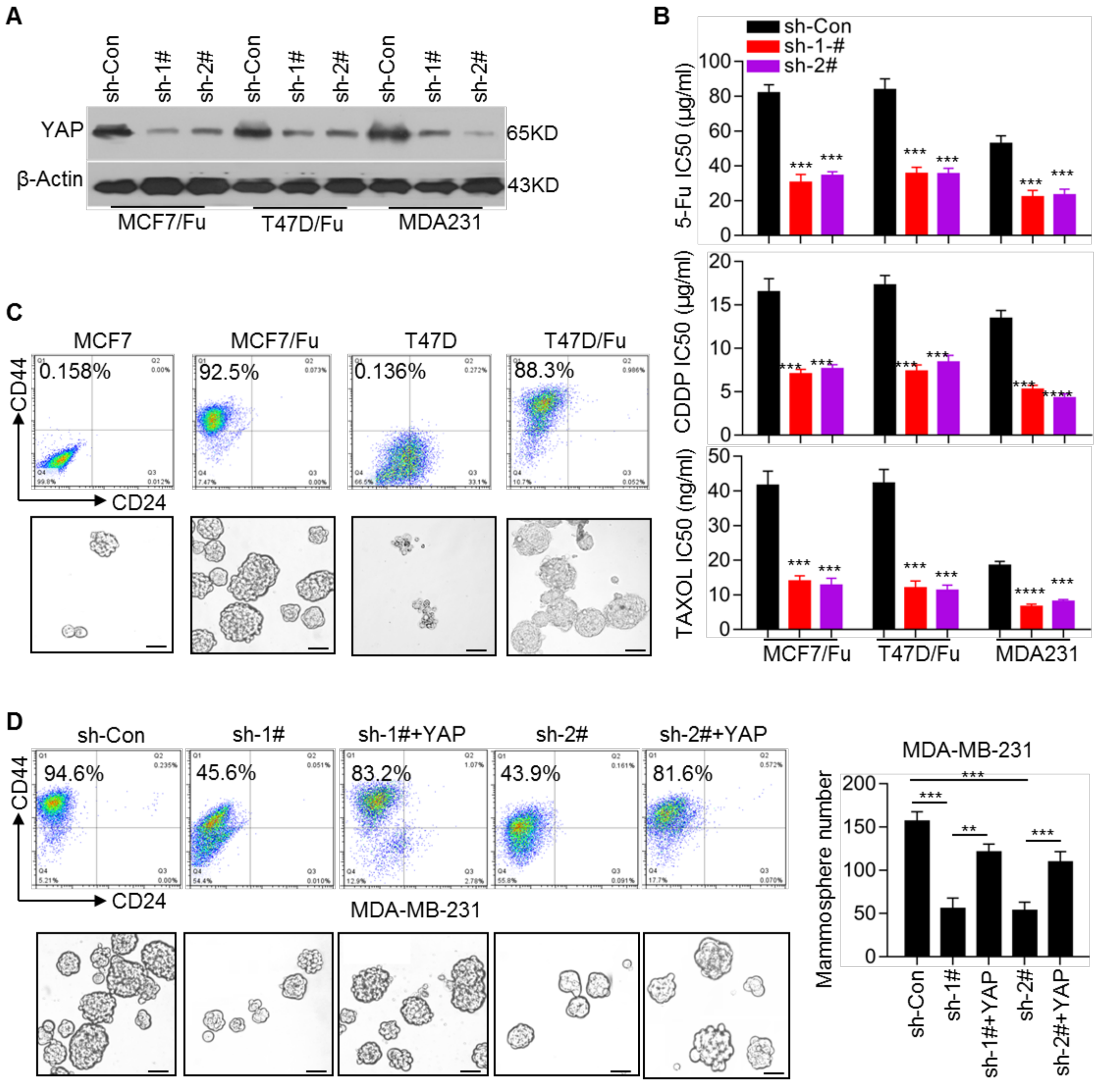

Figure S3, related to Figure 2. SRGN maintains breast cancer stem cell traits via activating YAP signaling.

(A) The protein level of YAP in cell lines with YAP knockdown was detected by western blot. (B) The IC50 values of drugs in cell lines with YAP knockdown were calculated from MTS assays. Student's t-test, Mean±s.d. (n=3), *** p < 0.001, **** p <

0.0001. (C) The CD44^{high}/CD24^{low} portion determined by flow cytometry and the ability of mammosphere formation was detected in cell lines. (D) The CD44^{high}/CD24^{low} portion determined by flow cytometry and the ability of mammosphere formation was detected in MCF-7/5-Fu cells with expression interference of SRGN and YAP. Scale bar, 100 µm. Student's t-test, Mean±s.d. (n=3), *** $p < 0.001$.

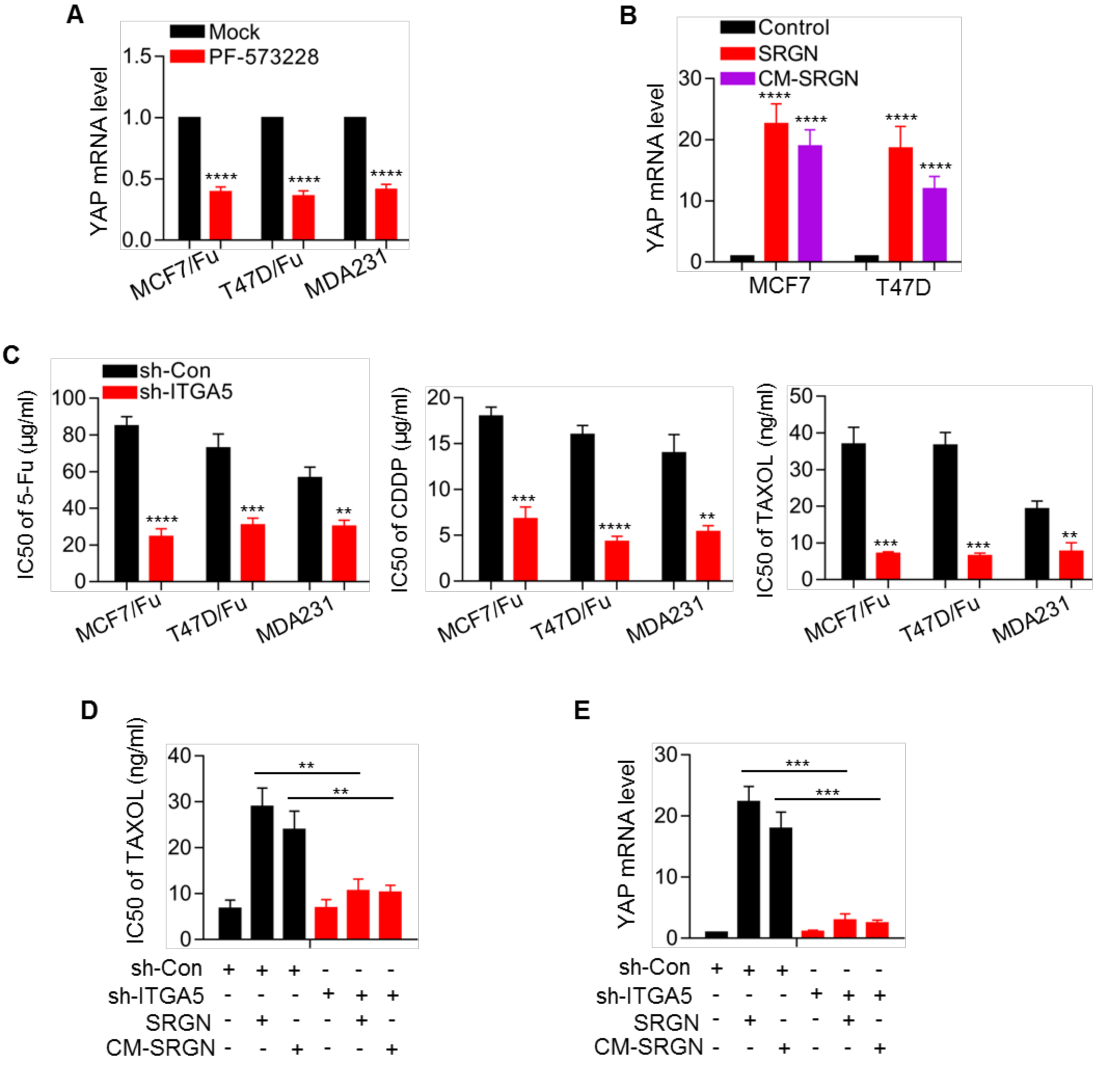

Figure S4, related to Figure 3. SRGN enhanced YAP expression via activating ITGα5/FAK/CREB signaling. (A) YAP mRNA expression in cell lines treated with FAK inhibitor was examined by qRT-PCR. (B) YAP mRNA expression in cell lines with SRGN knockdown was examined by qRT-PCR. Student's t-test, Mean±s.d.

(n=3), **** $p < 0.0001$. (C) YAP mRNA expression in cell lines with SRGN overexpression or incubated with CM-SRGN was examined by qRT-PCR. Student's t-test, Mean±s.d. (n=3), **** $p < 0.0001$. (D) The IC50 values of drugs in cell lines with ITGA5 knockdown were calculated from MTS assays. Student's t-test, Mean±s.d. (n=3), ** $p < 0.01$, *** $p < 0.001$, **** $p < 0.0001$. (E) The IC50 value of TAXOL in MCF-7 cells with expression interference of ITGA5 companied with SRGN overexpression or CM-SRGN incubation were calculated from MTS assays. Student's t-test, Mean±s.d. (n=3), ** $p < 0.01$. (F) YAP mRNA level in MCF-7 cells with expression interference of ITGA5 companied with SRGN overexpression or CM-SRGN incubation was examined using qRT-PCR. Student's t-test, Mean±s.d. (n=3), *** $p < 0.001$.

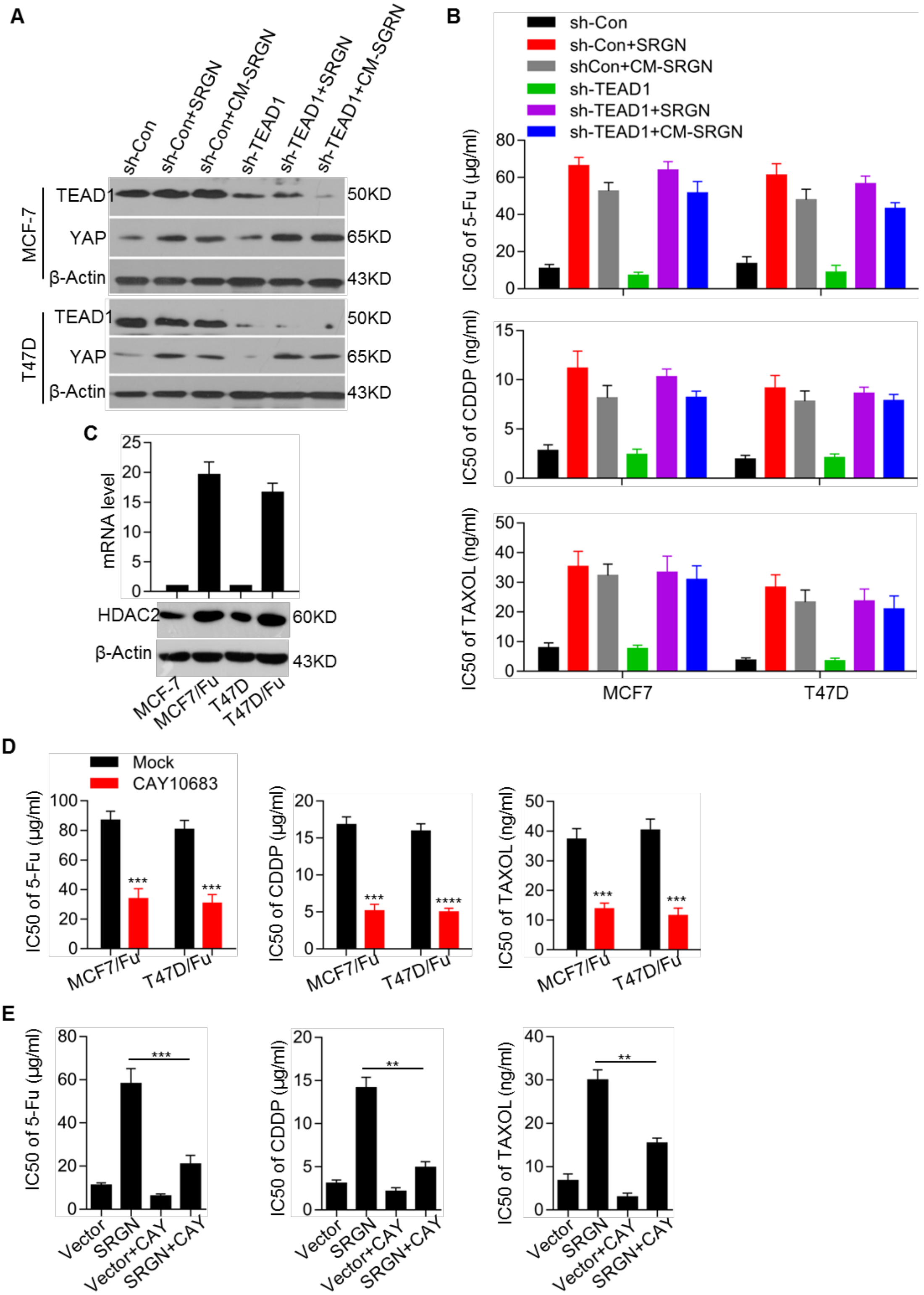

Figure S5, related to Figure 5. SRGN enhances HDAC2 expression to maintain the BC SCs traits. (A) Related protein levels were examined by western blot. (B) The IC50 values of drugs in cell lines with expression interference of SRGN and TEAD1 were calculated from MTS assays. (C) HDAC2 protein levels in selected cell

lines were detected by western blot. (D) The IC50 values of drugs in cell lines incubated with HDAC2 inhibitor were calculated from MTS assays. Student's t-test, Mean±s.d. (n=3), *** $p < 0.001$, **** $p < 0.0001$. (E) The IC50 values of drugs in MCF-7 cells with HDAC2 inhibitor CAY10683 and SRGN overexpression were calculated via MTS assays. Student's t-test, mean±s.d. (n=3), ** $p < 0.01$, *** $p < 0.001$.

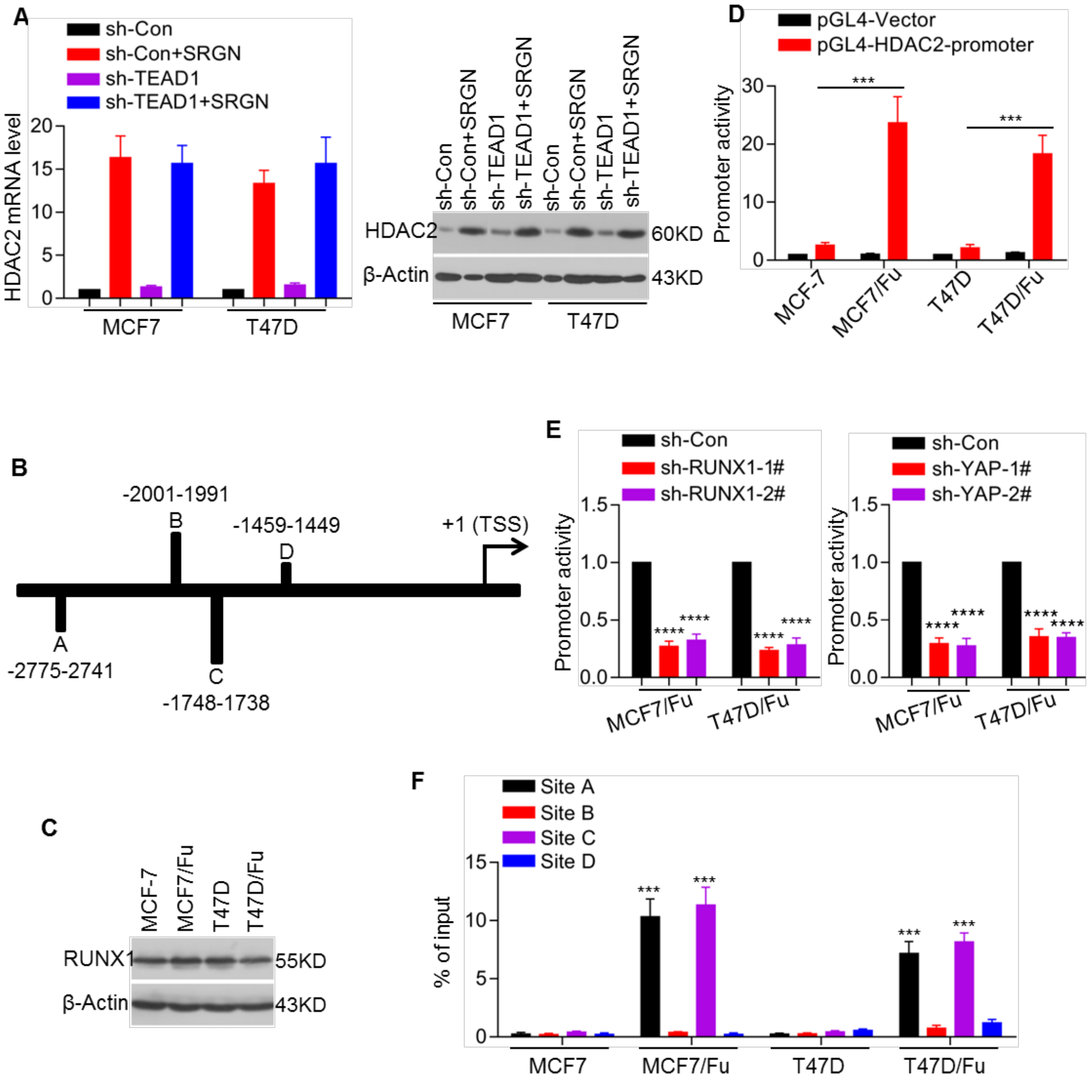

Figure S6, related to Figure 6. YAP interacts with RUNX1 to transcriptionally regulate HDAC2 expression in BC cells. (A) mRNA and protein levels in related cell lines with TEAD1 knockdown combined with SRGN overexpression were

examined by qRT-PCR and western blot respectively. (B) The putative RUNX1 binding site on the potential promoter region of *HDAC2* was predicted by online server analysis (http://jaspar.binf.ku.dk). (C) RUNX1 protein levels in cell lines were examined by western blot. (D) Luciferase activity in selected cell lines driven by HDAC2 promoter was determined by reporter assay. Student's t-test, Mean±s.d. (n=3), *** $p < 0.001$. (E) Luciferase activity driven by HDAC2 promoter in cell lines with RUNX1 knockdown or YAP knockdown was determined by reporter assay. Student's t-test, Mean±s.d. (n=3), *** $p < 0.001$. (F) The enrichment of YAP at HDAC2 promoter was determined by ChIP-qPCR. Student's t-test, Mean±s.d. (n=3), *** $p < 0.001$.

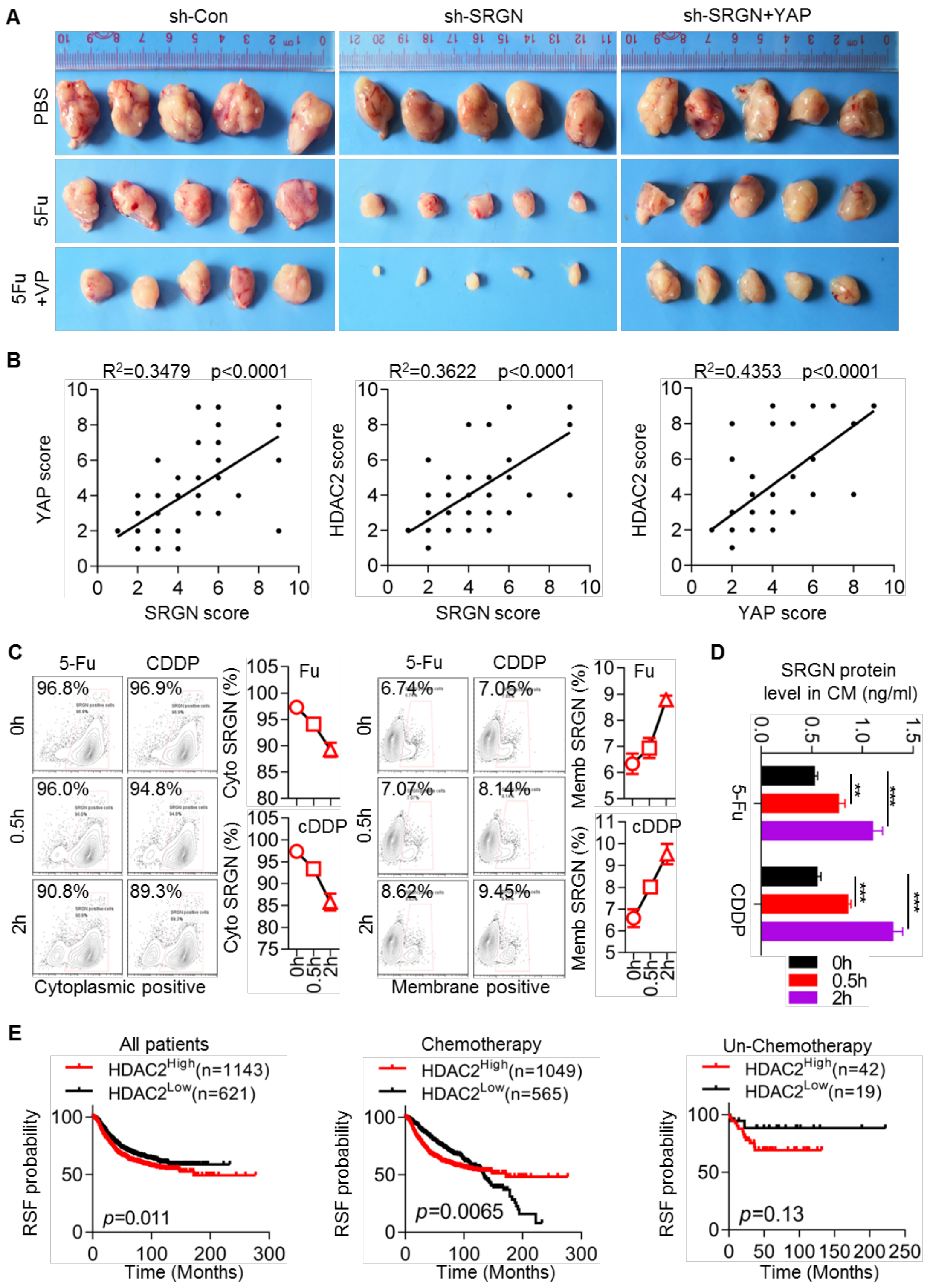

Figure S7, related to Figure 7. SRGN/YAP promotes chemo-resistance in vivo and correlates with poor outcome in BC patients. (A) Images of xenograft tumors formed by MDA-MB-231 cells with expression change of SRGN and YAP subcutaneously with or without chemotherapy. (B) The correlation between related protein level was analyzed using GraphPad Prism software. (C) The translocation of

SRGN protein upon chemotherapy was detected by flow cytometry analysis. (D) SRGN protein levels in CM upon chemotherapy were examined by ELISA assays. Student's t-test, Mean±s.d. (n=3), ** $p < 0.01$, *** $p < 0.001$. (E) Kaplan-Meier plots for HDAC2 were drawn in breast cancer cohorts from TCGA dataset. Log-rank p values were shown.

Supplemental Experimental Procedures

RNA extraction and quantitative real-time PCR analyses (qRT-PCR)

The TRIzol reagent was used to extract total RNA. cDNA was generated with the PrimeScriptRT reagent kit. GAPDH was performed as an internal reference for cytoplasmic gene expression. The expression change of genes was calculated by the $2^{-\Delta\Delta Ct}$ method.

MTS assay

Cells were seeded into 96-well plates, and cell viability upon drugs with different concentration was assessed by MTS assay using the CellTiter 96 Aqueous One Solution Cell Proliferation Assay, following the manufacturer's indications. Growth curves were plotted. IC50 values for drugs were calculated.

Western Blot

Total protein was extracted from cells using RIPA buffer (Thermo Scientific, Rockford, IL, USA) in the presence of protease inhibitors (Protease Inhibitor Cocktail, Thermo Scientific) after deglycosylation (1,2). The protein concentration of lysates was measured using a BCA Protein Assay Kit (Thermo Scientific). Equivalent amounts of protein were mixed with 5×Lane Marker Reducing Sample Buffer

(Thermo Scientific), and resolved by electrophoresis in a 10% SDS–polyacrylamide gel and then transferred onto Immobilon-P Transfer Membrane (Merck Millipore). The membranes were blocked with 5% non-fat milk in Tris-buffered saline and then incubated with the primary antibodies ollowed by secondary antibody. The signal was detected using enhanced chemiluminescence western blot detection kit. The primary antibody for SRGN (ab156991) was from abcam. The primary antibodies for YAP (#14074), FAK (#71433), p-FAK (#8556), CREB (#9197), p-CREB (#9198), ITGA5 (#98204), TEAD1 (#12292), HDAC2 (#57156) and RUNX1 (#4336) were from Cell Signaling Technology. The primary antibody for β-Actin (A5316) was from sigma.

ELISA assay

The SRGN concentration in the serum of breast cancer patients and the supernatant of serum-free cultured cells was measured for 48 h using SRGN ELISA Kit (CUSABIO, China) according to the manufacturer's instructions.

Immunofluorescence

Cells were grown on poly-L-lysine-coated glass coverslips (BD Biosciences, San Jose, CA), and then fixed with 4% paraformaldehyde, and permeabilized with PBS containing 0.1% Triton X-100 (PBS-T). Coverslips were incubated in blocking solution containing 2% BSA in PBS for 1 h, and incubated with the appropriate primary YAP antibody for 1 h at room temperature. After incubation with Alexa Fluor 594-conjugated (red) goat anti-rabbit (#R37117) secondary antibody (Thermo Scientific, Rockford, IL, USA), cells were stained with DAPI for nuclear staining and then visualized by fluorescence microscopy.

Mammosphere Assay

Mammosphere assays were performed as previously described (3). Briefly, single cell suspensions of cell lines were suspended at a density of 20,000 cells/mL in Dulbecco's modified Eagle's medium/F-12 containing 5 mg/mL insulin, 0.5 mg/mL hydrocortisone, 2% B27, and 20 ng/mL epidermal growth factor and seeded into six-well plates with ultra low-attachment surface (2 mL per plate). Mammospheres were counted after 1 to 2 weeks.

Flow Cytometry Analysis

The anti-CD44 (clone G44-26) and anti-CD24 (clone ML5) antibodies used for FACS analysis were obtained from BD Bioscience. Briefly, cells were incubated with trypsin–EDTA and dissociated. Cells were pelleted by centrifugation at 500 g for 5 minutes at 4°C, resuspended in 100 μL of monoclonal mouse anti-human CD24-PE antibody and a monoclonal mouse anti-human CD44-APC antibody, and incubated for 20 minutes at 4°C. The sorting was performed following the manufacturer's instructions.

Luciferase reporter assay

HDAC2 promoter was cloned into the pGL4-reporter vector upstream of the luciferase gene. Cells were seeded in 96-well plates and co-transfected with the pGL4-reporter vector and the pRL-TK Renilla luciferase vector using Lipofectamine 2000 (Invitrogen). After transfection of 48 h, luciferase activity was determined using a Dual-Luciferase Reporter Assay System (Promega) on the BioTek Synergy 2. To determine the direct read-out of YAP transactivity, the synthetic YAP/TAZ-responsive luciferase reporter (8XGTIIC-lux) was used (4). The Renilla luciferase activity was

used as internal control and the firefly luciferase activity was calculated as the mean ± SD after being normalized by Renilla luciferase activity.

ChIP-qPCR

The ChIP assay was performed using the EZ-CHIP™ chromatin immunoprecipitation kit (Merck Millipore). Briefly: Chromatin proteins were cross-linked to DNA by addition of formaldehyde to the culture medium to a final concentration of 1%. After a 10 min incubation at room temperature, the cells were washed and scraped off in ice-cold phosphate-buffered saline (PBS) containing Protease Inhibitor Cocktail II. Cells were pelleted and then resuspended in lysis buffer containing Protease Inhibitor Cocktail II. The resulting lysate was subjected to sonication to reduce the size of DNA to approximately 200–1000 base pairs in length. The sample was centrifuged to remove cell debris and diluted ten-fold in ChIP dilution buffer containing Protease Inhibitor Cocktail II. Samples were kept on ice at all times. A 5 μl sample of the supernatant was retained as "Input" and stored at 4°C. Then 5 μg of antibodies were added to the chromatin solution and incubated overnight at 4°C with rotation. After antibody incubation, protein G agarose was added and the sample incubated at 4°C with rotation for an additional 2 h. The protein/DNA complexes were washed with Wash Buffers four times and eluted with ChIP Elution Buffer. Cross-links were then reversed to free DNA by the addition of 5M NaCl and incubation at 65°C for 4 h. The DNA was purified according to the manufacturer's instructions. 50 μl of DNA was obtained for each treatment. 0.2 μl of DNA from each group was used as a template for qPCR. The results were calculated by normalizing to the input.

Co-immunoprecipitation

For the co-immunoprecipitation assay, the cells were lysed with modified TNE buffer (50 mM Tris [pH 8.0], 1% Nonidet P-40 [NP-40], 150 mM NaCl, 2 mM EDTA, 10 mM sodium fluoride, 10 mM sodium pyrophosphate) supplemented with 1 mg/L aprotinin, 1 mM sodium orthovanadate (Na_3VO_4) and 1 mg/L leupeptin. Lysates were centrifuged and cleared by incubation with 25 µl of Protein A/G gel for 1.5 hr at 4°C. The pre-cleared supernatant was subjected to IP using the first antibodies at 4°C overnight. Then, the protein complexes were collected by incubation with 30 µl of Protein A/G gel for 2 hr at 4°C. The protein complexes were resolved by SDS-PAGE. Subsequently, western blot was performed.

Immunohistochemistry

The sections were dried at 55°C for 2 h and then deparaffinized in xylene and rehydrated using a series of graded alcohol washes. The tissue slides were then treated with 3% hydrogen peroxide in methanol for 15 min to quench endogenous peroxidase activity and antigen retrieval then performed by incubation in 0.01 M sodium cirate buffer (pH 6.0) and heating using a microwave oven. After a 1 h preincubation in 10% goat serum, the specimens were incubated with primary antibody overnight at 4°C. The tissue slides were treated with a non-biotin horseradish peroxidase detection system according to the manufacturer’s instruction (DAKO, Glostrup, Denmark). Two different pathologists evaluated the immunohistological samples.

Primers

Primers for qRT-PCR

GAPDH

Forward: 5'-ATTCCATGGCACCGTCAAGGCTGA-3'

Reverse: 5'-TTCTCCATGGTGGTGAAGACGCCA-3'

SRGN

Forward: 5′-TCCAACAAGATCCCCCGTCT-3′

Reverse: 5′-TTCCGTTAGGAAGCCACTCC-3′

CDH1

Forward: 5'-GTCAGTTCAGACTCCAGCCC-3'

Reverse: 5'-AAATTCACTCTGCCCAGGACG-3'

IL-6

Forward: 5'-ACTCACCTCTTCAGAACGAATTG-3'

Reverse: 5'-CCATCTTTGGAAGGTTCAGGTTG-3'

SPHK1

Forward: 5'-GCTCTGGTGGTCATGTCTGG-3'

Reverse: 5'-CACAGCAATAGCGTGCAGT-3'

ESRP1

Forward: 5'-GCCAAGCTAGGCTCGGATG-3'

Reverse: 5'-CAGTCCTCCGTCAGTTCCAAC-3'

YAP

Forward: 5'-TAGCCCTGCGTAGCCAGTTA-3'

Reverse: 5'-TCATGCTTAGTCCACTGTCTGT-3'

CTGF

Forward: 5'-CAGCATGGACGTTCGTCTG-3'

Reverse: 5'-AACCACGGTTTGGTCCTTGG-3'

CYR61

Forward: 5'-CTCGCCTTAGTCGTCACCC-3'

Reverse: 5'-CGCCGAAGTTGCATTCCAG-3'

CYR61

Forward: 5'-ATGGCGTACAGTCAAGGAGG-3'

Reverse: 5'-TGCGGATTCTATGAGGCTTCA-3'

Primers for shRNAs

For SRGN

sh-1#: 5'-GCAAATTGGCAGGTAATATTT-3'

sh-2#: 5'-GGTAATATTTCATACCTAAAT-3'

For YAP

sh-1#: 5'-GGAAGCTGCCCGACTCCTTCT-3'

sh-2#: 5'-GCAGGTTGGGAGATGGCAAAG-3'

For ITGA5

sh-1#: 5'-GCTACCTCTCCACAGATAACT-3'

sh-2#: 5'-GCAGAGAGATGAAGATCTACC-3'

For TEAD1

sh-1#: 5'-GGATCAGACTGCAAAGGATAA-3'

sh-2#: 5'-GGGCTGATTTAAACTGCAATA-3'

For HDAC2

sh-1#: 5'-GCTTCTCTTGTATCCTCTACT-3'

sh-2#: 5'-GCTAGACTAGGTGAAATTAAG-3'

For RUNX1

sh-1#: 5'-GCACCCAGCAACGCCCATTTC-3'

sh-2#: 5'-GGATGCAACTCGCCCTGTTTG-3'

Primers for ChIP-qPCR

For SRGN promoter:

Site A

Forward: 5'-TATCGTAGATTGACTTTTAGGGAA-3'

Reverse: 5'-TATGCGTCCTTTGTTCCAGA-3'

Site B

Forward: 5'-TATTGATAGGAACTATTGTTTTGGT-3'

Reverse: 5'-CCAGAAGTGTGTCCTCCAAC-3'

Site C

Forward: 5'-CGTTCCTGATTTCTGGCTTATTC-3'

Reverse: 5'-TCAAGAACATAGGATTTTCAGTTACAA-3'

For HDAC2 promoter:

Site A

Forward: 5'-TTTGAGCAAGTAGGTGGATTAG-3'

Reverse: 5'-ACCTGAATAGCAACATCTAACAAT-3'

Site B

Forward: 5'-TCCTTAACTATGCACGCATCC-3'

Reverse: 5'-TGGGAGGAGTTTTATCCCATGA-3'

Site C

Forward: 5'-TGGTTTAAAATTATTAAATAATAAATAC-3'

Reverse: 5'-AATGGGGCAGTATTACTTGG-3'

Site D

Forward: 5'-ACACTCCATTCATAGTGGGACA-3'

Reverse: 5'-CAAAATCAACTTGGAAGATTCTGA-3'

References

1. Li XJ, Ong CK, Cao Y, Xiang YQ, Shao JY, Ooi A*, et al.* Serglycin is a theranostic target in nasopharyngeal carcinoma that promotes metastasis. Cancer Res **2011**;71(8):3162-72 doi 10.1158/0008-5472.CAN-10-3557.
2. Korpetinou A, Skandalis SS, Moustakas A, Happonen KE, Tveit H, Prydz K*, et al.* Serglycin is implicated in the promotion of aggressive phenotype of breast cancer cells. PLoS One **2013**;8(10):e78157 doi 10.1371/journal.pone.0078157.
3. Grimshaw MJ, Cooper L, Papazisis K, Coleman JA, Bohnenkamp HR, Chiapero-Stanke L*, et al.* Mammosphere culture of metastatic breast cancer cells enriches for tumorigenic breast cancer cells. Breast cancer research : BCR **2008**;10(3):R52 doi 10.1186/bcr2106.
4. Dupont S, Morsut L, Aragona M, Enzo E, Giulitti S, Cordenonsi M*, et al.* Role of YAP/TAZ in mechanotransduction. Nature **2011**;474(7350):179-83 doi 10.1038/nature10137.
